# Genomic analysis of novel *Yarrowia*-like yeast symbionts associated with the carrion-feeding burying beetle *Nicrophorus vespilloides*

**DOI:** 10.1186/s12864-021-07597-z

**Published:** 2021-05-03

**Authors:** Karina Brinkrolf, Shantanu P. Shukla, Sven Griep, Oliver Rupp, Philipp Heise, Alexander Goesmann, David G. Heckel, Heiko Vogel, Andreas Vilcinskas

**Affiliations:** 1 Department of Bioresources, Fraunhofer Institute for Molecular Biology and Applied Ecology, Ohlebergsweg 12, 35392 Giessen, Germany; 2 Bioinformatics and Systems Biology, Justus Liebig University Giessen, Heinrich-Buff-Ring 58, 35302 Giessen, Germany; 3 Department of Entomology, Max Planck Institute for Chemical Ecology, Jena, Germany; 4 Institute for Insect Biotechnology, Justus Liebig University Giessen, Heinrich-Buff-Ring 26-32, 35392 Giessen, Germany

**Keywords:** Ephemeral resources, Transcriptomics, Metabolic profiling, rDNA variability, Carrion beetles, Digestion, Detoxification, *Nicrophorus vespilloides*, *Yarrowia*

## Abstract

**Background:**

Mutualistic interactions with microbes can help insects adapt to extreme environments and unusual diets. An intriguing example is the burying beetle *Nicrophorus vespilloides*, which feeds and reproduces on small vertebrate carcasses. Its fungal microbiome is dominated by yeasts that potentially facilitate carcass utilization by producing digestive enzymes, eliminating cadaver-associated toxic volatiles (that would otherwise attract competitors), and releasing antimicrobials to sanitize the microenvironment. Some of these yeasts are closely related to the biotechnologically important species *Yarrowia lipolytica*.

**Results:**

To investigate the roles of these *Yarrowia*-like yeast (YLY) strains in more detail, we selected five strains from two different phylogenetic clades for third-generation sequencing and genome analysis. The first clade, represented by strain B02, has a 20-Mb genome containing ~ 6400 predicted protein-coding genes. The second clade, represented by strain C11, has a 25-Mb genome containing ~ 6300 predicted protein-coding genes, and extensive intraspecific variability within the ITS–D1/D2 rDNA region commonly used for species assignments. Phenotypic microarray analysis revealed that both YLY strains were able to utilize a diverse range of carbon and nitrogen sources (including microbial metabolites associated with putrefaction), and can grow in environments with extreme pH and salt concentrations.

**Conclusions:**

The genomic characterization of five yeast strains isolated from *N. vespilloides* resulted in the identification of strains potentially representing new YLY species. Given their abundance in the beetle hindgut, and dominant growth on beetle-prepared carcasses, the analysis of these strains has revealed the genetic basis of a potential symbiotic relationship between yeasts and burying beetles that facilitates carcass digestion and preservation.

**Supplementary Information:**

The online version contains supplementary material available at 10.1186/s12864-021-07597-z.

## Background

Insects are evolutionarily highly successful organisms in terms of biodiversity. Their diversification and colonization of new and sophisticated ecological niches is promoted by their ability to form mutualistic interactions with other organisms, including bacteria, fungi, protozoa, plants and even other insects. Mutualistic microbes play particularly important roles in insect development, nutrition and reproduction, for example by producing digestive enzymes, antibiotics and essential nutrients, and by removing toxins and protecting the host against parasites [1]. One example for such a mutualistic relationship between insects and their symbionts are aphids. They are exclusive phloem sap feeders and have been able to occupy this nutrient deficient niche because bacterial symbionts provide essential amino acids [2]. Aside from symbiotic interactions with bacteria, also fungal symbionts are found in insects, with relatively well-studied examples being ants, termites and ambrosia beetles [3–5]. The presence of yeasts has been reported in many species of Coleoptera, Diptera, Homoptera, Hymenoptera, Isoptera and Lepidoptera, but their contribution to host fitness has only been investigated in a few cases [6]. Their coevolution can result in interactions where insects such as ambrosia beetles mediate spread of yeasts, or in relationships where the two cannot exist without each other [7–9]. For example, the removal of yeast symbionts from the brown planthopper *Nilaparvata lugens* reduces egg and larval survival, and prevents the completion of ecdysis [10]. In addition to the symbiotic relationships between yeasts and insects, their coevolution also provides examples for the development of entomopathogenic yeasts such as Ophiocordyceps [11].

Here we investigated yeasts isolated from the burying beetle *Nicrophorus vespilloides* (family Silphidae), a necrophagous species found in northern America, Europe and the Palearctic that uses the carcasses of small vertebrates as a food source and breeding site [12]. Breeding adults discover fresh carcasses based on volatile emissions and bury them to hide the carcass from competing insects, mammals and birds. Carcass burial involves a sophisticated preservation strategy, in which a breeding pair removes hair/feathers [13], covers the surface with anal and oral exudates, and rolls the carcass into a ball [14]. The female lays eggs near the carcass and both parents make additional preparations such as providing entry points for the newly hatched larvae. Following burial, carcasses prepared by burying beetles do not show typical signs of decomposition, such as bloating or the emission of strong odors associated with putrefying meat [15].

The preservation of carcasses by *N. vespilloides* involves the regulation of bacterial and fungal communities, thus providing larvae with an optimal diet for development [16]. The microbial communities of prepared carcasses contain microorganisms found in the *N. vespilloides* gut, and both the bacterial and fungal communities of tended carcasses are distinct from those of decomposing carcasses that have not been associated with beetles. Beetle-prepared carcasses produce lower levels of toxic nitrogenous waste compounds such as putrescine, and also show lower levels of protease activity compared to untended, decomposing carcasses [16]. Given that many of these metabolites are generated by microbial degradation, carrion preservation is probably facilitated by the suppression of parasitic and competitive microorganisms following inoculation with mutualistic gut microbes. It is therefore likely that carrion preservation involves the cooperation between the host insect and its microbiome [17]. The secretions of adult and larval burying beetles, which are smeared on carcasses by the tending beetles, contain antimicrobials that inhibit Gram-positive and Gram-negative bacteria as well as fungi [18]. *N. vespilloides* produces a diverse range of antimicrobial peptides (AMPs), some of which have sex-specific and/or carrion-dependent expression profiles [19, 20] as well as differential expression between gut regions, suggesting that the gut microbiome may be curated to facilitate carcass utilization [17]. Adult beetles may sanitize the carcass by releasing antimicrobial volatiles such as phenols, amides, alcohols and fatty acids [18].

Adult *N. vespilloides* beetles host a conserved and characteristic microbiome including species representing the bacterial orders Xanthomonadales, Lactobacillales, Clostridiales, Enterobacteriales and Neisseriales [15–17, 21]. The adult gut also contains bacterial symbionts that produce nematicidal compounds to control phoretic nematodes [22]. The fungal microbiome is dominated by yeasts, some of which (closely related to the important industrial species *Yarrowia lipolytica*) are particularly abundant in the adult hindgut and anal secretions, as well as in larvae [16]. The transcriptomic analysis of prepared carcasses revealed that the same *Yarrowia*-like yeast (YLY) strains are metabolically active on the carcass and express genes involved in carbohydrate, lipid and protein metabolism, and the biosynthesis of vitamins and sterols [16, 17]. YLY strains appear on carcasses following the initiation of carcass preparation by adult burying beetles but are not present in the surrounding soil, or in the native microbial communities of the carcass, indicating that the yeasts are transmitted by adult beetles via the carcass surface to offspring [15]. The cultivation of these YLY strains in vitro revealed the synthesis and secretion of fatty acids (oleic, myristic, palmitic and stearic acids) that are also detected in the anal and oral secretions that adult beetles apply to carcasses [18]. The YLY strains associated with burying beetles are therefore likely to contribute to carcass preservation.

Phylogenetic analysis of partial 28S rRNA genes showed that YLY strains associated with *N. vespilloides* are genetically diverse. They form two distinct clades with 91 to 98% identity to known *Y. lipolytica* strains based on similarity of the LSU gene [17]. *Yarrowia* is a genus of aerobic, non-pathogenic yeasts present in dairy and meat products, with the ability to produce large quantities of lipase and to utilize fatty acids, alkanes, alcohols and acetate [23, 24]. The discovery of novel YLY strains in burying beetles therefore not only provides an interesting opportunity to study the role of mutualistic yeasts in burying beetle ecology but could also provide new leads for efficient industrial processes. Here we sequenced and analyzed the genomes of five diverse YLY strains associated with *N. vespilloides* followed by transcriptomic and phenotypic (metabolic) characterization to investigate their roles in digestion, detoxification and carrion preservation.

## Results

### Genome sequencing and assembly

**Fig. 1:**
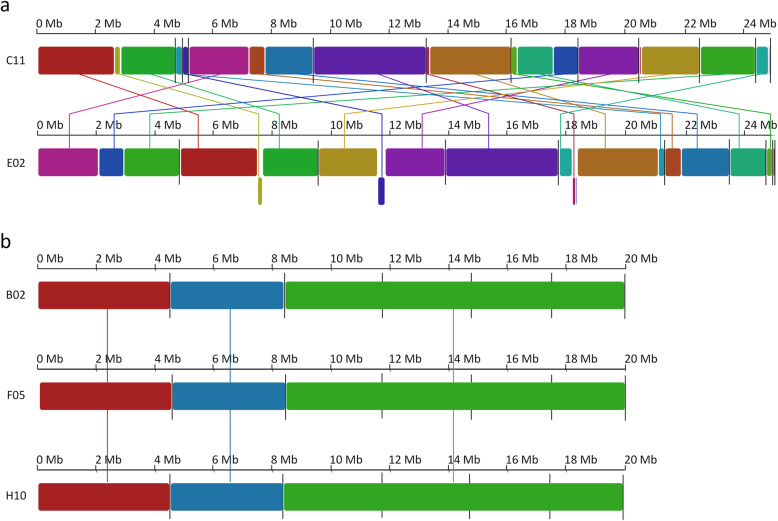
Sequence alignment of *Yarrowia*-like yeast genomes. Homologous regions are depicted in the same colors with connecting lines. Contig ends are indicated by black vertical lines. **a** Clade I genomes of strains C11 and E02. The comparison shows major sequence rearrangements between the two genomes. However, both genomes generally comprise the same building blocks. **b** Clade II genomes of strains B02, F05 and H10. All three genomes have an overall similar structure. No sequence rearrangements or additional sequences were detected

We selected five YLY strains from our previous study [17], namely strains C11 and E02 representing clade I and strains B02, F05 and H10 representing clade II isolated from *N. vespilloides* hindgut secretions. Whole-genome sequencing using PacBio technology produced 150,000–200,000 reads per strain with an N50 read length of up to 20,948 nt (Table 1). De novo assemblies of strains C11 and E02 indicated a genome size of ~ 25 Mb, represented by 11 and 9 contigs, respectively (Table 1). In contrast, the assemblies of strains B02, F05 and H10 indicated a genome size of ~ 20 Mb consisting in each case of six genomic contigs. For comparison, *Y. lipolytica* (currently the only *Yarrowia* species with a nuclear genome assembled to the chromosome level) has a genome size of 20.3–20.5 MB distributed across six chromosomes [25–27]. To investigate within-clade similarities among the YLY genomes, we determined the degree of synteny for the chromosomal loci in each clade separately. The alignment of strains C11 and E02 revealed several rearrangements between the two genomes (Fig. 1**a**). At the sequence level, this was accompanied by 143,978 sequence variations (SNPs and indels). In contrast, the alignment of strains B02, F05 and H10 showed no rearrangements, indicating that the genomes were highly conserved (Fig. 1**b**). At the sequence level, this was also reflected by the low number of variations between the strains, with 387 variations between strains B02 and F02, and 344 between strains B02 and H10.

**Table 1:** Sequencing and assembly statistics of *Yarrowia*-like yeast genomes

Feature	Clade I genomes		Clade II genomes		
	C11	E02	B02	F05	H10
Sequenced bases	1,980,785,767	2,372,271,037	2,585,237,711	2,853,963,881	2,190,534,112
Number of reads	150,243	173,939	185,386	202,765	181,738
N50 read length	18,953	19,592	20,539	20,948	17,012
Chromosomal contigs	11	9	6	6	6
Genome size total [Mb]	24.97	25.05	19.98	20.02	19.93

### Gene finding and functional annotation

We used RNA-Seq data generated for strain C11 (clade I) and strain B02 (clade II) to support gene finding for all five YLY strains. RNA samples for sequencing were prepared from cells maintained under different growth conditions to maximize the total number of transcripts covered. Gene finding identified 6319–6325 protein coding genes for clade I and 6452–6459 (~ 2% more) for clade II (Table 2). Given the difference in genome size between the two clades this equated to a coding density of 39.7% for clade I and 50.3% for clade II (Table 3, Additional File: Fig. S**1**). The clade I genomes featured longer intergenic regions (59.7% of the genome) than clade II (46.4% of the genome), which was primarily responsible for the larger genome size of strains C11 and E02. Thereby, the greater proportion of intergeneric regions in these genomes is due to the fact that some regions are significantly longer. For the clade I genome, e.g., 25% of the bases are located in intergenic regions with a length of 10 to 100 kb, while for the clade II genomes only 3% of the intergenic regions are that long (Additional File: Fig. S**2**). The difference in region numbers can also be shown for the introns, although a significant difference in intronic bases cannot be observed. The recently published improved genome sequence of *Y. lipolytica* CLIB89 [26] consists of 49.0% coding regions and 48.3% intergenic regions, which is closer in distribution to the clade II genomes B02, F05 and H10 than the clade I genomes C11 and E02 (Table 3).

**Table 2:** Gene finding and annotation statistics of *Yarrowia*-like yeast genomes

Feature	Clade I genomes		Clade II genomes		
	C11	E02	B02	F05	H10
Protein coding genes	6325	6319	6458	6459	6452
Transcripts	6374	6380	6549	6548	6543
Single-exon transcripts	4901	4903	5035	5050	4952
Multi-exon transcripts	1473	1477	1514	1498	1591
Transcripts with EC number	1652	1652	1700	1704	1696
Transcripts with eggNOC funcats	2444	2446	2548	2550	2536
Transcripts with high-confidence annotations	3368	3373	3444	3444	3438
Hypothetical transcripts	1000	1008	1069	1062	1053
tRNAs	525	521	566	566	566
rRNAs	139	141	123	132	117

**Table 3:** Ratio of exons, introns and intergenic regions in *Yarrowia*-like yeast genomes

Strain	Genome size [Mb]	Total gene length [Mb]	Total exon length [Mb]	Coding regions [%]	Total intron length [Mb]	Intron regions [%]	Total length of intergenic regions [Mb]	Intergenic regions [%]
**C11**	24.88	10.65	9.91	39.81	0.74	2.98	14.24	57.21
**E02**	25.05	10.60	9.90	39.53	0.70	2,79	14.45	57.69
**B02**	19.98	10.71	10.05	50.31	0.65	3.27	9.28	46.42
**F05**	20.02	10.71	10.06	50.25	0.66	3.28	9.30	46.47
**H10**	19.93	10.71	10.04	50.37	0.67	3.35	9.22	46.28
**CLIB122**	20.55	9.73	9.44	45.91	0.30	1.44	10.82	52.65
**W29/CLIB89**	20.55	10.51	10.08	49.03	0.44	2.14	10.03	48.83

**Fig. 2:**
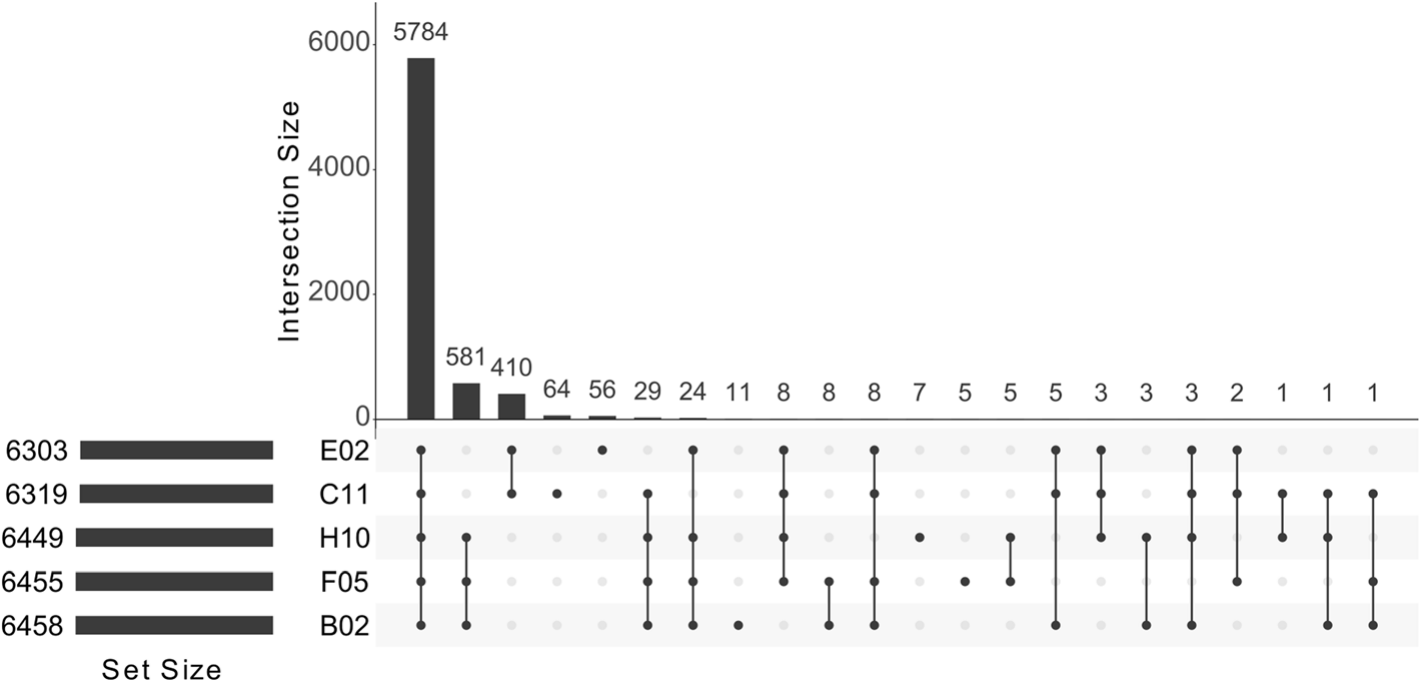
Number of reciprocal best BLAST hits between subsets of the five *Yarrowia*-like yeast genomes. Plots were visualized using R package UpSetR [28] The core genome of all five YLY strains includes 5784 genes (90.3%). Clade I genomes share 410 genes and the clade II genomes share 581 genes. Aside from the core genome, no other subset of the diagram includes as many genes as the clade specific subsets. This indicates that genes in the clade I genomes C11 and E02 are more closely related to each other than the other genomes, which is also true for the three clade II genomes B02, F05 and H10. Furthermore, this comparison shows that clade I genomes (C11, E02) have more strain-specific genes than the clade II genomes (B02, F05, H10). For a detailed analysis of subsets of different YLY genomes also see **Additional File: Fig. S****3**

We used an automated genome annotation pipeline for the functional annotation of the YLY genomes. Annotations were assigned to 53.4% of the genes and EC numbers were assigned to 26.3% (Table 2). The core genome of all five strains featured 5784 genes (Fig. 2). Other genes were clade-specific, with 410 genes present in strains C11 and E02 but lacking homologs in B02, F05 or H10, and 581 genes present in strains B02, F05 and H10 but lacking homologs in C11 or E02 (Fig. 2). Based on comparisons between subsets of genomes and the number of genes shared between the YLY strains, we found that (i) the number of strain-specific genes in the clade I genomes C11 and E02 was ~ 10-fold higher than the number of strain-specific genes in the clade II genomes B02, F05 and H10, indicating that the clade I genomes show greater diversity at the gene level (Additional File: Fig. S3a and b); (ii) there are always more genes shared between genomes within a clade than between genomes of different clades (Fig. 2, underlined numbers and Additional File: Fig. S**3**c); and (iii) all five YLY genomes (clades I and II) differ significantly from the closely-related *Y. lipolytica* CLIB122 genome (Additional File: Fig. S**3**d and e).

The core genome and strain-specific genes were identified using reciprocal best BLAST hits, but the absence of a reciprocal hit may lead to the inaccurate classification of singletons. We therefore calculated the exact number of singletons for each of the YLY stains because these might represent adaptations in the yeast genomes either due to their association with beetles or because they are carrion-colonizing specialists. We identified eight and nine singletons in strains C11 and E02, respectively, but none in strains B02 and F05, and only one in strain H10 (Additional File: Table S**1**). For strain C11, all eight singletons were annotated as hypothetical proteins, seven of which were also represented by homologs in strain E02. Interestingly, these homologs were not annotated as genes in strain E02 either due to SNPs that cause frameshifts or due to larger indels. Among the nine singletons in strain E02, six were unique to this strain (four of which were homologous to retroviral-related Pol polyproteins from opus) and three were represented by homologous genes in C11 (Additional File: Table S**2**). The only clade II singleton (in strain H10) was annotated as two short hypothetical proteins, whereas one long open reading frame spanned the corresponding region in genomes B02 and F05.

### Phylogenetic diversity of the *Yarrowia*-like strains at the rDNA level

The internal transcribed spacer (ITS) region, including ITS1, 5.8S and ITS2 or the D1/D2 region of the rRNA large subunit (LSU), are often used for taxonomic classification by identifying SNPs differing with respect to a reference genome. We used the sequences of primers ITS1 and ITS4 [29] to localize the ITS region and the sequences of primers NL1 and NL4 [30] to identify the D1/D2 region of the LSU. These regions are directly linked and form a ~ 880 bp segment that was present in all five YLY genome assemblies, albeit with different copy numbers (B02 = 3, C11 = 9, E02 = 9, F05 = 8 and H10 = 1) (Additional File: Table S**3**). These rDNA copies were always located at contig ends and were sometimes arranged as repeats (Additional File: Fig. S**4**). Considering the sequencing depth of these genomic regions for the single strains, we also calculated the expected rRNA copy numbers from our data, and predicted 4.7 copies for strain B02, 12.5 copies for C11, 9.8 copies for E02, 4.8 copies for F05 and 7.4 copies for H10.

**Fig. 3:**
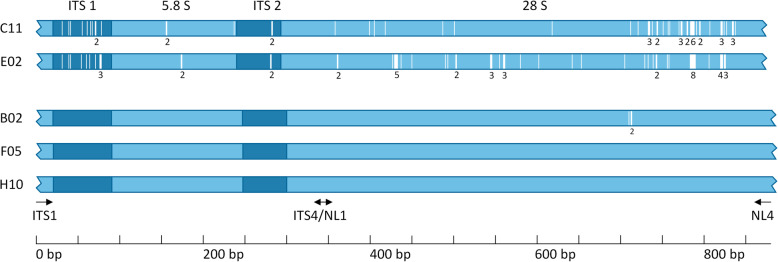
Intraspecific variable base positions within the ITS–D1/D2 regions of the single *Yarrowia*-like yeast genomes. Assemblies of the five genomes analyzed in this study contain different copy numbers of the ITS–D1/D2 regions (B02 = 3, C11 = 9, E02 = 9, F05 = 8, H10 = 1). To identify intraspecific variability among the rRNA copies, the genomes were analyzed separately without cross-species comparisons. Strains C11 and E02 showed high intraspecific variability of the assembled rRNA copies. In contrast, of the clade II strains B02, F05 and H10, only B02 displayed any intraspecific variability with three variable positions. Blue bars represent the sequence alignments of the ITS–D1/D2 region for each strain separately with the variable sequence positions marked as white bars. Numbers beneath the white bars are the numbers of adjacent variable positions. Positions of the primers that determine the ITS–D1/D2 region are shown as black arrows

We determined the intraspecific variability between these copies in genomes B02, C11, E02 and F05, and observed high variability for the ITS-D1/D2 region in the clade I genomes C11 and E02, with 58 and 63 variable positions respectively (Fig. 3). These included SNPs as well as single-base insertions and deletions (indels). In contrast, the ITS-D1/D2 regions of the clade II genomes B02, F05 and H10 did not show this variability: all rDNA copies were identical within and between the three genomes in the clade, with the exception of one copy in strain B02, which featured a deletion of three nucleotides at the 3′ end of the sequence (Fig. 3).

Despite the high intraspecific variability within the ITS–D1/D2 regions of strains C11 and E02, we used all the available sequences to find the minimal distance between these two strains and the other three (Table 4). We found no sequence differences between the two most similar ITS–D1/D2 copies in strains C11 and E02, suggesting that both strains probably represent the same species. Given the number of sequence variants, we concluded that the two strains in clade I and the three in clade II are likely to represent different species, both of which appear to be novel and differ from the industrial yeast *Y. lipolytica* (Table 4). Additional BLASTn analysis of the ~ 880 bp ITS-D1/D2 regions against NCBI’s nt revealed that strains B02, F05 and H10 are most closely related to *Y. osloensis* (91.9% ID) and *Y. lipolytica* (91.6% ID) on the rRNA level. Analyzing the different rRNA variants of YLY strains C11 and E02, we found IDs of 94.2–95.5% (C11) and 90.9–95.7% (E02) with *Y. osloensis*, 92.2–93.3% (C11) and 89–93.1% (E02) with *Y. deformans* and 91.3–93.1% (C11) and 88.5 and 92.3% (E02) with *Y. lipolytica*.

**Table 4:** Minimal number of sequence variances between ITS–D1/D2-regions of *Yarrowia*-like yeasts

Strain	C11	E02	B02	F05	H10	*Y. lip* CLIB89
**C11**	0	0	67	67	67	52
**E02**	0	0	70	70	70	69
**B02**	67	70	0	0	0	75
**F05**	67	70	0	0	0	75
**H10**	67	70	0	0	0	75
***Y. lip*** **CLIB89**	52	69	75	75	75	0

For genomes with multiple sequence variants (C11, E02, B02) BLAST analysis was carried out for all possible combinations. The table summarizes the results of the best BLAST hits, i.e. the minimal number of variations assumed between two strains

**Fig. 4:**
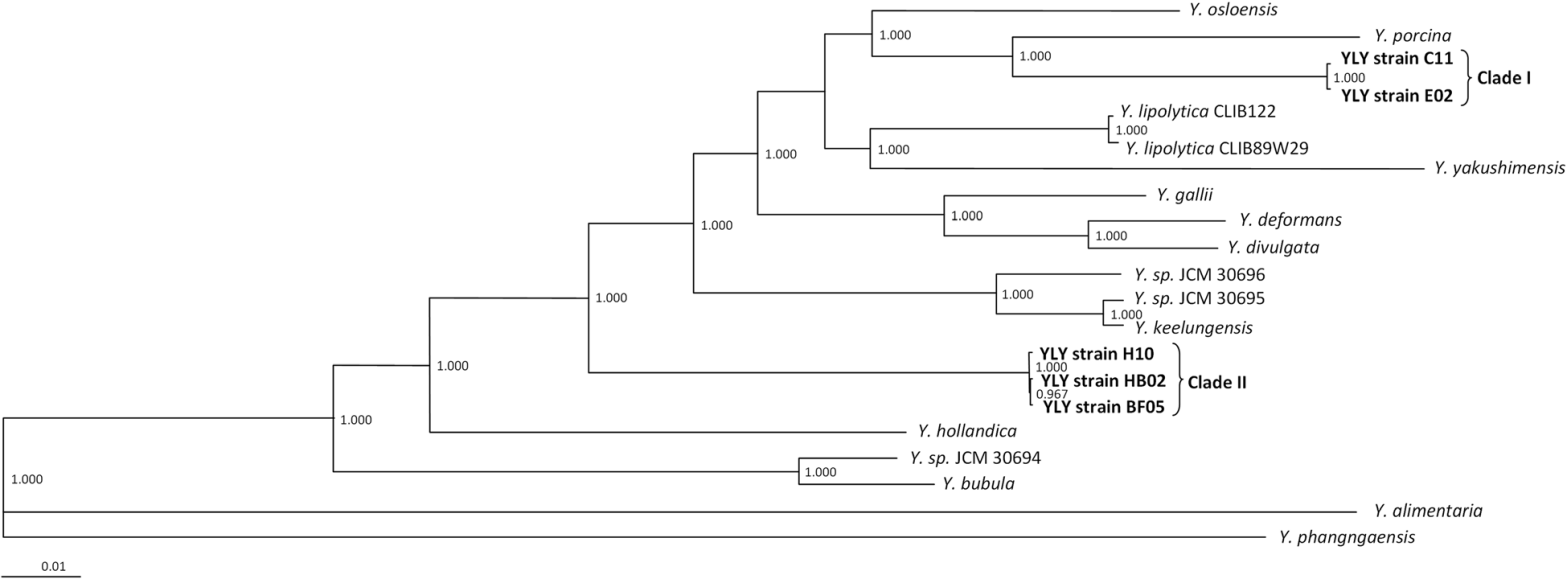
Phylogenetic tree of *Yarrowia*-like yeasts. The tree was constructed in EDGAR [31] based on the amino acid sequences of the “*Yarrowia* core genome” using FastTree 2.8.1 with default settings. The core genome includes the common set of genes shared by *Y. yakushimensis*, *Y. porcina*, *Y. phangngaensis*, *Y. osloensis*, *Y. hollandica*, *Y. galli*, *Y. divulgata*, *Y. deformans*, *Y. bubula*, *Y. alimentaria*, *Yarrowia* sp. JCM 30695, *Yarrowia* sp. JCM 30696, *Yarrowia* sp. JCM 30694, *Y. keelungensis* and 2 *Y. lipolytica* strains. This gene set is composed of 3542 genes. The five yeast strains we investigated are highlighted in bold. The scale displays substitutions per site. Local branch support values were computed with the Shimodaira-Hasegawa test [32]

We conducted further phylogenetic analysis based on the core genome of the five YLY strains and all other sequenced and assembled *Yarrowia*-like genomes available thus far: *Y. yakushimensis*, *Y. porcina*, *Y. phangngaensis*, *Y. osloensis*, *Y. hollandica*, *Y. galli*, *Y. divulgata*, *Y. deformans*, *Y. bubula*, *Y. alimentaria*, *Yarrowia* sp. JCM 30695, *Yarrowia* sp. JCM 30696, *Yarrowia* sp. JCM 30694 and *Y. keelungensis*, as well as two representative strains of *Y. lipolytica*. This core genome included 3542 genes. The resulting clusters supported our previous interpretations, with the five YLY strains clearly separated into two different clusters. The clade I strains C11 and E02 were most similar to *Y. porcina* and *Y. osloensis* and showed greater similarity to *Y. lipolytica* than the clade II strains B02, F05 and H10 (Fig. 4).

### Mitochondrial genome analysis

**Fig. 5:**
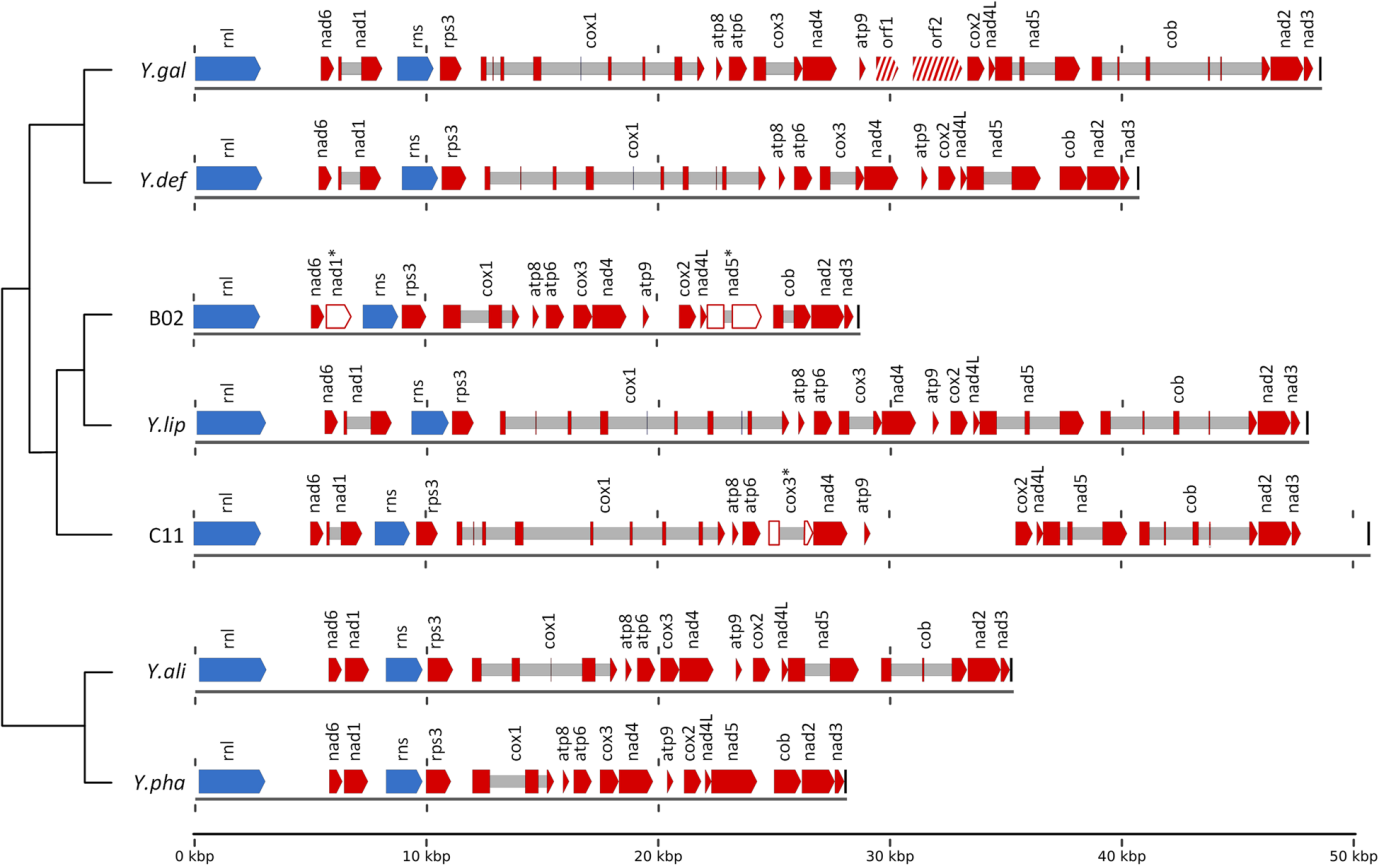
Schematic maps of *Yarrowia*-like yeast mitochondrial genomes. Blue arrows represent rRNA genes; red arrows represent protein-coding genes. Red rectangles and arrows connected by gray bars indicate genes with an exon-intron structure, where introns are gray. Shaded red arrows represent open reading frames not present in any of the other mitochondrial genomes. White arrows with red outline represent regions homologous to genes, but due to indels or SNPs these genes are not functional (marked with *). The end of each mitochondrial contig is indicated by a black bar. In case of splice variants, the longest version is displayed. Phylogenetic relation was calculated on the amino acid level based on the mitochondrial core genome using EDGAR and is indicated on the left. Lengths of the branches in this tree have no significance

In addition to the nuclear genome, the mitochondrial genome was assembled into a single contig for each of the five YLY strains. For strains C11 and E02, the size of the mitochondrial genome was ~ 50.6 kb with a G + C content of 20.3%, whereas the mitochondrial genome of strains B02, F05 and H10 was smaller (~ 28.6 kb) with a G + C content of 23.3% (Table 5). Although *Y. lipolytica* is the only *Yarrowia* species with a genome sequence completed to the chromosomal level, several YLY mitochondrial genomes have been sequenced in addition to *Y. lipolytica*, namely *Y. phangngaensis*, *Y. alimentaria*, *Y. deformans*, and *Y. galli* [33]. Based on the published YLY mitochondrial genome sequences, we identified homologous genes/regions using BLAST in all five strains, including seven subunits of the NADH:ubiquinone oxidoreductase complex (complex I) and the typical genes encoding respiratory chain complexes III, IV and V (Fig. 5). Although homologous regions were present, we noted that the *nad1* and *nad5* genes in strain B02 and the *cox1* gene in strain C11 appear non-functional due to nonsense mutations (Fig. 5). The C11 and E02 mitochondrial genomes contained 17 introns, representing a coding density of 36.6% coding bases (63.4% non-coding bases). In contrast, the smaller B02, F05 and H10 mitochondrial genomes contained only four introns, representing a coding density of 44.3% coding bases (54.7% non-coding bases).

**Table 5:** Assembly statistics for mitochondrial (mt) genomes of *Yarrowia*-like yeast strains

Feature	Clade I mt-genomes		Clade II mt-genomes		
	C11	E02	B02	F05	H10
Mitochondrial genomes [nt]	50,623	50,627	28,621	28,658	28,527
G + C content [%]	20.30	20.29	23.30	23.31	23.26
Number of protein coding mt-genes	13*	14	12*	11*	13*
Number of mt-introns	17	17	4	4	4
Total length of mt-introns [nt]	14,073	14,074	2846	2564	2487

*We identified homologous regions matching all 14 protein coding genes in the mitochondrial reference genomes of YLYs, but some of these regions are non-functional due to sequence variations and nonsense mutations

We calculated a phylogenetic tree based on the mitochondrial core genome using EDGAR. This tree confirms the close relationship between *Y. deformans* and *Y. gallii* and between *Y. alimentaria* and *Y. phangngaensis*, as already shown at the chromosomal level (Figs. 4 and 5). In contrast, for the YLYs analyzed here, the phylogeny of the mitochondria does not fully reflect the chromosomal phylogeny. While the clade I strain C11 clusters with *Y. lipolytica* at the chromosomal level, the clade II strain B02 is closer to *Y. lipolytica* when comparing the mitochondria.

### Phenotypic characterization

We used Biolog Phenotype MicroArrays (PM plates) to determine the ability of strains C11 and B02 to utilize diverse nutrient substrates and survive under different growth conditions. Among the carbon sources provided in the PM plates (PM 1A and 2B), both strains were able to utilize arabinose, mannose, ribose, fructose, glycerol, and organic acids including succinic acid, malic acid, butyric acid, pyruvic acid, fumaric acid, acetic acid, citric acid, and propionic acids. None of the strains utilized sucrose, cellobiose, pectin, or galactose. Among the nitrogen sources provided in the PM plates (PM plate 3B), both strains were able to utilize ammonia, urea, uric acid, agmatine and allantoin. None of the strains utilized nitrite or nitrate. Both strains utilized putrescine as a carbon and nitrogen source. Both strains used acetamide as a nitrogen source but not as a carbon source. For the PM1 plates (carbon sources), strain B02 successfully utilized 31.25% of the carbon-based substrates, compared to 25% for strain C11. The proportion of utilized substrates did not differ significantly between the two strains (test of equal proportions, X-squared = 0.64, *p* = 0.42). There was no difference in the ability of each strain to utilize plate PM3 nitrogen sources (B02 = 57.2%, C11 = 56.2%; test of proportions, X-squared < 0.0001, *p* = 1.0).

Both strains were able to grow in the presence of up to 6% NaCl, and utilized creatine, glycerol and glutathione in the presence of 6% NaCl. Both strains were also able to utilize up to 5% sodium sulfate, 10% ethylene glycol, 6% sodium formate, 4% urea, 3% sodium lactate, 50 mM sodium phosphate and 50 mM ammonium sulfate. Both strains showed broad pH tolerance (growth in the pH range 4.0–9.5). The strains also utilized all 19 of the provided amino acids at pH 9.5 (only cysteine was not tested). At pH 4.5 strain B02 was able to utilize all 18 of the provided amino acids (tyrosine and cysteine were not tested) whereas strain C11 was only able to utilize glutamic acid, isoleucine, leucine, methionine, phenylalanine and valine. Both strains utilized urea, creatine, putrescine, and cadaverine at pH 9.5, indicating their potential for diverse nitrogen metabolism under highly alkaline conditions.

### Analysis of specific genetic pathways

As potential mutualists, we hypothesize that YLY can help *N. vespilloides* to break down complex nutrients in the carrion, thereby providing larvae and adults with nutrition and/or facilitating digestion and detoxification. In order to identify antimicrobial peptides or antibiotics within the genetic repertoires of the five YLY strains, we compared all the gene sets to the publically-available databases APD [34], DRAMP [35] and CAMP [36], but did not generate any hits. We also analyzed all genomes with the fungal version of antiSMASH [37] and identified one non-ribosomal peptide synthetase (NRPS) gene cluster in each strain, which might contribute to the symbiotic relationship with *N. vespilloides*. We also analyzed four specific pathways: putrescine degradation, uric acid recycling, triglyceride digestion and protein digestion.

**Fig. 6:**
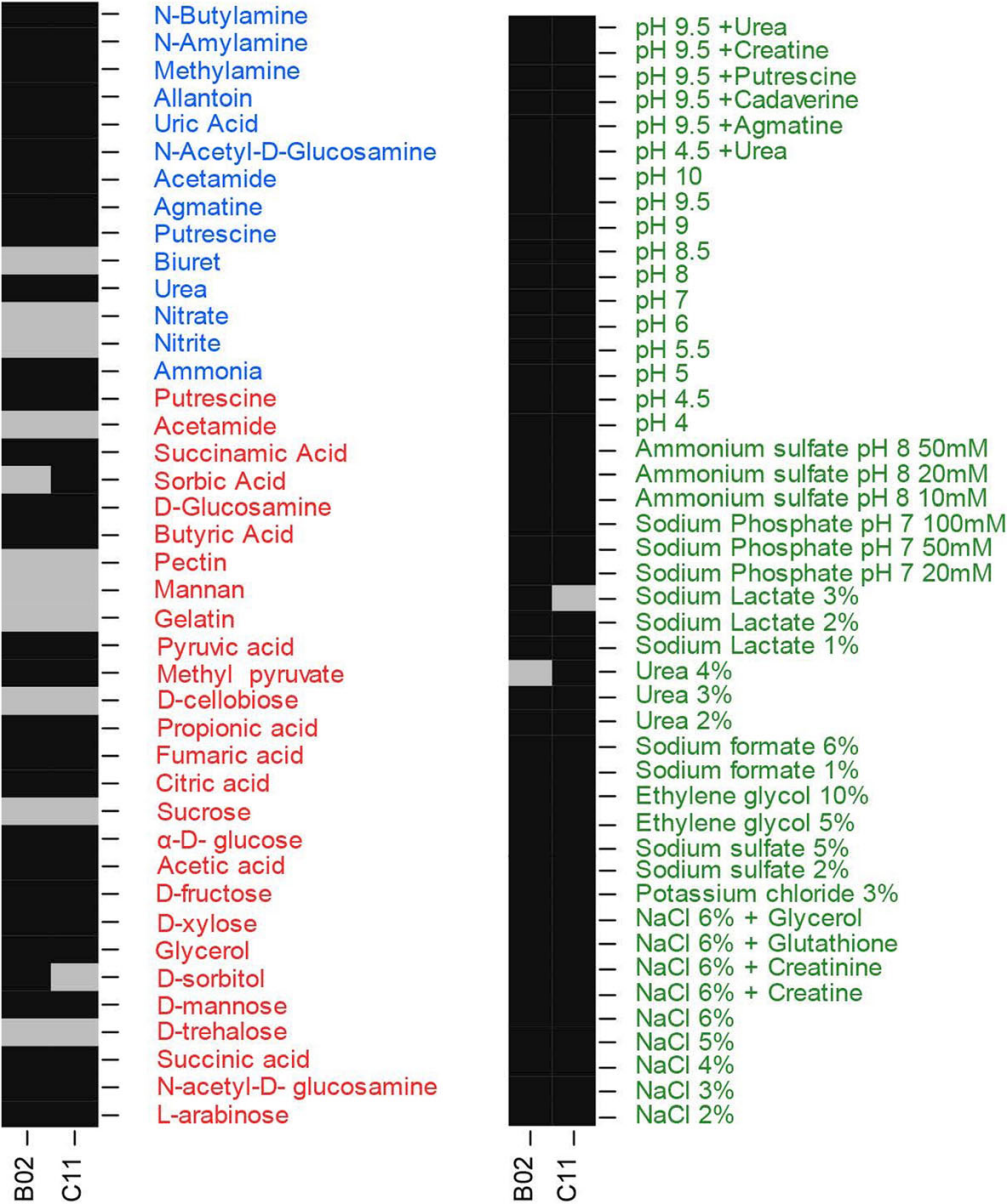
Substrate utilization assays using Biolog Phenotype Microarrays for strains C11 and B02. Black shading indicates utilization of the substrate based on threshold score values whereas gray shading indicates lack of utilization. Carbon sources (MicroPlates PM1 and PM2A) are indicated in red, nitrogen sources (MicroPlates PM3B) are indicated in blue, osmolytes (MicroPlates PM9 9) and pH conditions (MicroPlates PM10) are indicated in green

**Fig. 7:**
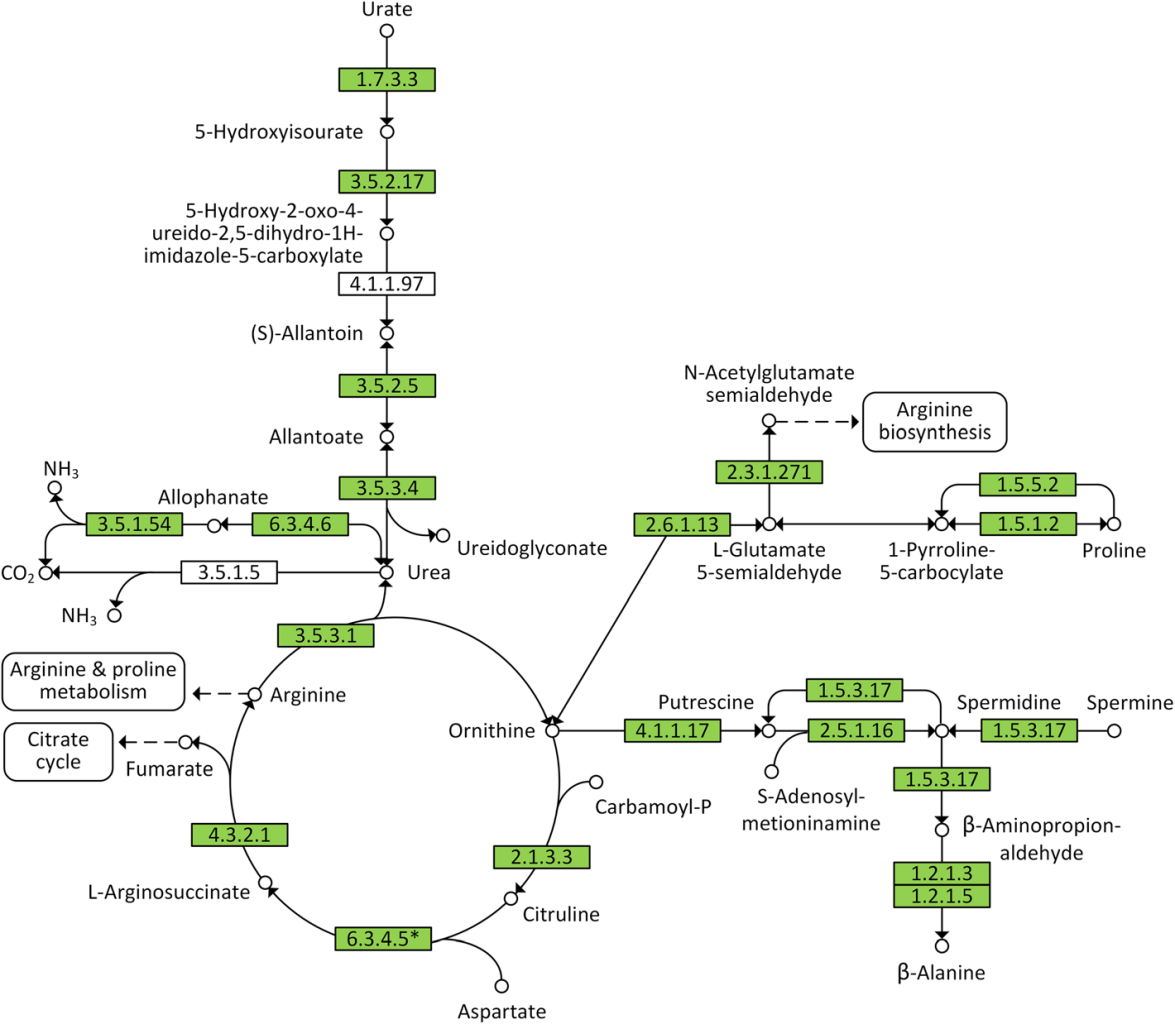
Metabolic pathways of putrescine degradation and uric acid recycling in strains C11 and B02. Green boxes represent EC numbers of enzymes annotated for strains C11, B02 and *Y. lipolytica*. White boxes represent EC numbers of enzymes that are not annotated. EC 6.3.4.5* is annotated for strains C11 and B02 but is not present in *Y. lipolytica*. This figure was constructed using information provided by KEGG pathway maps 00220, 00230, 00330 and 00410 [38]

Putrescine, a toxic biogenic amine produced by bacteria during carrion decomposition, is responsible for the characteristic odor associated with meat decay. Carcasses prepared by *N. vespilloides* show negligible amounts of putrescine in the feeding cavity of tended carcasses [15, 16]. Furthermore, the PM assays described above revealed that both C11 and B02 can utilize putrescine. We therefore screened the C11 and B02 genomes for genes involved in the detoxification of putrescine. We found that both strains possess a gene encoding putrescine aminopropyltransferases (EC 2.5.1.16) that convert putrescine to spermidine (Figs. 6 and 7). The genomes and transcriptomes of both strains also contained sequences that converts putrescine into β-alanine via spermidine and spermine through the arginine and proline metabolism pathway (Fig. 7). Similarly, we identified sequences representing genes required for the enzymatic recycling of uric acid (urate) to ammonia via allantoin, allantoate and urea. The conversion of urea to ammonia is most likely carried out via allophanate synthesis because the enzyme urease (which directly converts urea to ammonia) was not detected in either strain (Fig. 7). We also identified genes representing the enzymatic pathway that converts the triglycerides in animal carcasses into glycerol, which involves the production of extracellular lipases (equivalent of the *Y. lipolytica* extracellular lipase LIP2). We found that strains B02 and C11 also produce alkaline proteases and acid proteases, equivalent to the extracellular proteases found in *Y. lipolytica* [39, 40].

## Discussion

The ability of insects to survive under extreme conditions and to adapt to diets that are difficult to utilize is often mediated by microbial symbionts [1]. Such relationships between insects and fungal symbionts that help to degrade dietary plant-derived materials are known in fungus-growing termites [5] and passalid beetles that ingest wood in the presence of xylose-fermenting gut yeasts [41]. The burying beetle *N. vespilloides* also hosts YLY strains in its hindgut which are deposited on the carrion during its preparation as a food source and nesting site.

Accordingly, we carried out the genomic and functional characterization of five YLY strains that appear to represent strains of two distinct clades of YLYs in the core microbiome of the burying beetle as previously described by [15, 17] and used PM assays to determine their metabolic capabilities. Analysis of the functionally annotated draft genomes of all five strains revealed genome sizes of ~ 25 Mb for strains C11 and E02 (clade I) and ~ 20 Mb for strains B02, F05 and H10 (clade II). The clade II genomes were most similar in size to the *Y. lipolytica* genome, which ranges from 20.1 to 20.6 Mb depending on the strain (accession numbers NC_006067, HG934059 and CP017553). Two other *Yarrowia* species in the NCBI genome database are *Y. deformans* (accession number BCIW01000000) and *Y. keelungensis* (accession number BCJD01000000) with genome sizes of 20.9 and 21.8 Mb, respectively. The similar genome sizes of our clade II strains and *Y. lipolytica* result in a similar coding density, whereas the coding density of the clade I strains C11 and E02 was much lower. Whole-genome comparisons showed that the three clade II strains (B02, F05 and H10) were similar in genome structure and sequence, whereas strains C11 and E02 featured several large rearrangements as well as many indels and SNPs. All five strains were dissimilar to *Y. lipolytica*, with core genome analysis revealing that only 88% of protein-coding genes were conserved between strain C11, strain B02 and *Y. lipolytica*. Each strain in this comparison also featured ~ 400 genes that were not shared with the other two, supporting the hypothesis that none of the strains we investigated belongs to the species *Y. lipolytica*.

Taxonomic classification is often based on the comparison of short conserved DNA sequences. In fungi, the ITS region and LSU domains 1 and 2 (D1/D2) are often used for this purpose, and species-level resolution is conventionally based on the divergence of these regions by less than 1% [42]. For strains C11 and E02 we observed high intraspecific variability within the ITS–D1/D2 region which was not observed in strains B02, F05 and H10. Given the presence of multiple paralogs in both C11 and E02, we used all available sequences to calculate the minimal distance between the strains. This analysis suggested that the clade I and II strains are likely to represent distinct species, with a minimum sequence divergence of ~ 8% between clades and < 1% within clades when the most similar ITS–D1/D2 regions were compared. However, the multiple divergent copies of ITS–D1/D2 in strains C11 and E02 makes it unclear whether these are two strains of a single species or alternatively two separate species. We were unable to compare the ITS–D1/D2 region of other *Yarrowia* species, because the whole-genome sequences of *Y. lipolytica* and other *Yarrowia*-like species in the NCBI genome database do not include this region.

As a complementary approach, we also compared the ITS–D1/D2 sequences to fungal sequences available in rDNA databases. One disadvantage of this approach is that the entries in these databases are usually generated by PCR followed by direct sequencing, which leads to the loss of intraspecific variability unless multiple amplicons for each strain are sequenced in parallel. Using available rDNA sequence data from *Y. lipolytica*, we found that our five YLY strains clearly differ from *Y. lipolytica* with minimal sequence distances of 8.5% for the three clade II strains, 7.7% for strain E02 and 6.9% for strain C11. BLAST analysis of the single ITS-D1/D2 copies of the five strains against NCBI’s nt database revealed that strains C11 and E02 are most closely related to *Y. osloensis* and *Y. deformans* with varying percentage of similarity depending on the copy used. Strains of clade II are most closely related to *Y. osloensis* and *Y. lipolytica* on the rRNA level. To verify whether sequence similarities on the rRNA level are also mirrored by the genomes on a global scale, we constructed a core genome-based phylogenetic tree including several *Yarrowia*-like genomes. Interestingly, this global analysis confirmed a close taxonomic relation between the clade I strains C11 and E02 with *Y. osloensis*, but it also showed that both strains are almost similar among each other with regard to the core genome, despite the variances detected for the single ITS-D1/D2 copies. The clade II strains B02, F05 and H10 represent an early-emerging position in the phylogenetic tree with respect to a clade that contains *Y. lipolytica.* These findings supported our hypothesis that strains C11 and E02 probably belong to the same species, and that the clade I and II strains probably represent independent species within the genus *Yarrowia*.

Individual strains representing each clade (C11 for clade I and B02 for clade II) were tested for their metabolic capabilities on PM plates. Both strains metabolized diverse carbon and nitrogen sources over a broad pH range and when exposed to high salt concentrations. *Yarrowia* species generally colonize substrates such as carrion, meats, dairy products, sewage and oil-polluted environments, thus showing a preference for specialized niches rich in lipids and proteins [23, 24]. Both strains were able to metabolize amino acids, ammonia, urea, and putrescine, which could be important for carrion digestion and detoxification. The ability of both strains to tolerate pH extremes (pH 4–10) and up to 6% NaCl reflects their ability to withstand the diverse conditions found in the burying beetle gut and the carcass microenvironment, where YLY strains are abundant and metabolically active [17]. However, several hydrophobic substrates that *Yarrowia* species are known to utilize, including fatty acids, triglycerides, and other hydrocarbons [23, 24] were not tested in the current phenotypic microarray tests. Given the ability of burying beetles to produce their own digestive enzymes, AMPs and lysozymes to sanitize and utilize carcasses [16–18], the broad environmental tolerance of the YLY strains indicates how they can manage a free-living existence outside the beetles and yet evolve resistance to host defenses (AMPs and organic compounds such as phenols, amines, and fatty acids). The ability of YLY to survive in harsh environments shaped by the ability of adult beetles to suppress antagonists could simultaneously create a selective environment that promotes the growth and survival of YLY strains.

Our combined data provide strong evidence that the five YLY strains we characterized represent at least two species that form part of the core microbiome of the burying beetle, essential for the utilization of carcasses as an ephemeral but nutrient-rich diet. Using these vertically transmitted YLY strains, the beetles have partially outsourced the production of digestive enzymes, the sanitation of the carcass by antimicrobial volatiles [14, 17], and the degradation of carcass-associated odors to deter competitors such as fly maggots.

## Conclusions

Microbes can help insects to adapt to extreme environmental conditions and unusual diets. The fungal core microbiome of the burying beetle *Nicrophorus vespilloides* is dominated by yeasts, some of which are closely related to the biotechnologically important species *Yarrowia lipolytica*. We selected five of these YLY strains that were reported to represent strains of two distinct clades [17] for whole-genome sequencing. We found that strains of the two clades differ, e.g., in genome size and genome structure, and share clade specific subsets of genes. Phenotypic microarray analysis revealed that YLY strains from both clades utilized a diverse range of carbon and nitrogen sources, including microbial metabolites associated with putrefaction. Furthermore, they can grow in environments with extreme pH and salt concentrations. Our results suggest that the five YLY strains potentially represent new species. Given their abundance in the beetle hindgut, and dominant growth on beetle-prepared carcasses, the analysis of these strains has revealed the genetic basis of a potential symbiotic relationship between yeasts and burying beetles that facilitates carcass digestion and preservation.

## Methods

### Yeast isolation and genomic DNA extraction

A previous study by Shukla et al. isolated YLYs from the hindgut secretions of *N. vespilloides* larvae and pupae, which represent the first generation derived from beetles originating from wild populations collected in Germany near Giessen, as described in [17]. We selected five representative strains belonging to the two previously-identified YLY clades (strains C11 and E02 from clade I and strains B02, F05 and H10 from clade II) [17]. All five strains were grown in yeast extract/malt extract (YEME) broth (0.3% yeast extract, 0.3% malt extract, 1% dextrose, 0.5% peptone) and were incubated at 25 °C for 24–30 h, shaking at 250 rpm. Genomic DNA was extracted using the Qiagen Genomic Tip kit (Qiagen, Hilden, Germany) and the Qiagen genomic DNA buffer set according to the manufacturer’s protocol, with the following modifications. The cells were washed twice in 5 mL TE and resuspended in 5 mL Qiagen Buffer G2 containing 300 μg Qiagen RNase and 2 mg proteinase K. The resulting lysate was incubated at 50 °C for 120 min before centrifugation (5000×*g*, 4 °C, 10 min) and the supernatant was purified on a Qiagen 100/G genomic prep tip. Eluted DNA was precipitated by adding 3.5 mL isopropanol to 5 mL of eluate. The DNA was washed twice in 70% (v/v) ethanol, air dried and resuspended in 250 μL TE by slowly dissolving overnight at 4 °C. DNA quality was assessed by measuring the absorbance at 260/280 and 260/230 nm on a Nanodrop ND-1000 photometer (Thermo Fisher Scientific, Waltham, MA, USA). DNA integrity was confirmed by 0.8% agarose gel electrophoresis, with GeneRuler 1 kb Plus DNA Ladder molecular weight standards (Thermo Fisher Scientific).

### Genome sequencing and assembly

SMRTbell libraries (PacBio RS libraries with 8–12 kb inserts) were generated with size-selected DNA using the Blue Pippin system (Sage Science, Beverly, MA, USA). For strain H10, PacBio sequencing was carried out with P4 − C2 chemistry (Pacific Biosciences, Menlo Park, CA, USA) on six smart cells. All other strains were sequenced with P6 − C4 chemistry on six smart cells using a PacBio RSII instrument according to the manufacturer’s protocols.

Genome assemblies were prepared using the Hierarchical Genome Assembly Process Pipeline (HGAP.3) including read correction, Celera-based assembly, and assembly polishing with Quiver [43]. Small contigs completely included within other contigs and/or with significantly lower coverage than the large contigs were removed from the draft genomes. Contigs of strain H10 were ordered with CONTIGUator [44] using strain F05 as the reference to generate supercontigs. We identified mitochondrial contigs using the *Y. lipolytica* mitochondrial genome sequence as a query [45]. Sequence duplications at mitochondrial contig ends were identified by self-alignment using NUCmer [46]. Circularization of mitochondrial contigs was achieved with Circlator [47] based on the PacBio sequencing reads.

### YLY cultivation on different carbon and nitrogen sources

Two representative YLY strains (B02 and C11) were grown on different carbon and nitrogen sources in preparation for transcriptome analysis to characterize gene expression profiles involved in the utilization of different substrates. The transcriptomic response on different carbon sources was determined by growing cells separately in media containing glucose (2%), glycerol (3%) or stearin (1%) as the major carbon source. In addition, the media were supplemented with 0.5% casamino acids (AMRESCO, Solon, OH, USA) and 0.67% yeast nitrogen base without amino acids, carbohydrates and ammonium sulfate (US Biological, Salem, MA, USA). We also tested uric acid (0.15%) as alternative nitrogen sources, supplemented with glucose (2%) and 0.67% yeast nitrogen base without amino acids, carbohydrates and ammonium sulfate as above. Additionally, tryptic soy broth (1.7% tryptic digest of soy casein, 0.3% peptic digest of soy casein, 0.5% NaCl, 0.25% K_2_HPO_4_, 0.25% glucose) was used as a complex nitrogen growth medium. Diverse growth substrates were chosen in order to broaden yeast transcriptomic responses to the growth conditions and to gain further insights into their role in carrion digestion and detoxification. Stearin was tested to characterize the metabolic response to the digestion of triglycerides, which are abundant in carrion. Since burying beetles would excrete protein rich diets through uric acid, it was chosen as the sole nitrogen source in one of the growth media. Similarly, tryptic soy broth provided a complex, nitrogen-rich growth medium, which the yeasts are likely to encounter in the beetles’ gut or on beetle-prepared carcasses.

Glycerol stocks of the yeast strains were revived and pre-cultures were incubated in each of the media described above at 30 °C for 24 h, shaking at 150 rpm. From each pre-culture, 200 μL of the culture was transferred to 10 mL fresh medium and incubated at 30 °C, shaking at 250 rpm. The cultures were sampled regularly until the optical density at 600 nm (OD600) reached 0.5–0.7. We then centrifuged 1 mL of each culture (1200×g, 4 °C, 2 min) and the cell pellets were frozen in liquid nitrogen. Total RNA was extracted from the pellets by adding 300 μL TRIsure (Bioline, Luckenwalde, Germany). The mixtures were homogenized using a TissueLyser (Qiagen) for 5 min, and extracted with 150 μL bromo-3-chloropropane. The DNA was digested using Turbo DNase (Ambion) for 30 min at 37 °C. RNA was purified using RNA Clean & Concentrator (Zymo Research Europe, Freiburg, Germany) and RNA integrity and quality were assessed using the Agilent 2100 Bioanalyzer (Agilent Technologies, Waldbronn, Germany) with RNA Nano chips (Agilent Technologies). The RNA concentration was estimated using a Nanodrop ND-1000 photometer.

### Transcriptome sequencing and read mapping

Transcriptome sequencing was carried out by GATC Biotech (Konstanz, Germany) on an Illumina HiSeq2500 Genome Analyzer platform using paired-end (2 × 125 bp) read technology (HiSeq Rapid Run 125 bp PE) for the 10 YLY samples (two strains, each on three carbon and two nitrogen sources), yielding 20–25 million reads per sample. Quality control and mapping were carried out using CLC Genomics Workbench v9.1 (https://www.clcbio.com) [17, 48]. Sequencing reads were cleaned and trimmed to remove adapters and low-quality reads. Passing reads were aligned to the predicted coding sequences of strains B02 or C11 using the subread aligner implemented in CLC Genomics Workbench. BAM (mapping) files were then analyzed using QSeq (DNAStar, Madison, WI, USA) followed by sequence counting to estimate expression levels, using previously described parameters for read mapping and normalization [17, 48].

### Gene finding and functional annotation

Coding DNA sequences were predicted by combining AUGUSTUS [49] and BRAKER [50]. First, AUGUSTUS was implemented with (i) a pre-computed training dataset of *Y. lipolytica* included in AUGUSTUS, (ii) RNA-Seq data as hints, and (iii) a previously described iterative alignment step (https://bioinf.uni-greifswald.de/bioinf/wiki/pmwiki.php?n=IncorporatingRNAseq.Tophat). The RNA-Seq data from strain C11 were mapped to the chromosomal genomes of strains C11 and E02 to generate files of the corresponding hints. RNA-Seq data from strain B02 were then mapped to the genomes of strains B02, F05 and H10. Mappings were calculated using STAR [51]. Second, BRAKER was implemented using RNA-Seq data as hints. Predicted coding sequences from both approaches were compared using BEDtools [52] to identify genes predicted uniquely with BRAKER. This sub-list of genes with all child features was added to the AUGUSTUS annotation. The final set of genes for each strain includes all coding sequences from the AUGUSTUS approach for each strain supplemented with the gene sets uniquely identified with BRAKER. We predicted tRNAs using tRNAscan-SE [53] and rRNAs using RNAmmer v1.2 [54]. Chromosomal genomes were uploaded to the genome annotation platform GenDBE [55] for automated functional annotation using Blast2p to screen the databases SwissProt [56], InterPro [57], Pfam [58], SignalPeuk [59] and TIGRFAMs [60].

### Comparison of genome structures and gene repertoires

Chromosomal genomes were aligned using progressiveMAUVE [61]. Single-nucleotide polymorphisms (SNPs), insertions and deletions (indels) were revealed using the SMRTanalysis Quiver tool (Pacific Biosciences). For clade I genomes, whole-genome sequencing reads from strain E02 were mapped to the genome of strain C11. For clade II genomes, reads from strains F05 and H10 were mapped to the genome of strain B02. Distributions of coding and non-coding regions were calculated for the genomic positions of exons and introns from the gene finding data, and the coding density was visualized using DensityMap [62]. The core genome and singletons were defined by uploading the chromosomal genomes of the YLY strains to EDGAR (efficient database framework for comparative genome analysis using BLAST score ratios) [31]. For the core genome and singletons, we restricted calculations to only one transcript per gene (and genome), retaining that transcript of a gene, with the highest number of genomes containing a homolog of this transcript.

A phylogenetic tree was constructed in EDGAR on the basis of the “*Yarrowia* core genome” choosing the FastTree option for approximately-maximum-likelihood phylogenetic trees. This core genome includes all *Yarrowia* species fully sequenced and available from the NCBI GenBank by November 2019: *Y. yakushimensis* strain CBS 10253 (GCA_900518995.1), *Y. porcina* strain CBS 12935 (GCA_900519025.1), *Y. phangngaensis* strain CBS 10407 (GCA_900519005.1), *Y. osloensis* strain CBS 10146 (GCA_900519015.1), *Y. hollandica* strain CBS 4855 (GCA_900519065.1), *Y. galli* CBS 9722 (GCA_900519055.1), *Y. divulgata* CBS 11013 (GCA_900519045.1), *Y. deformans* strain CBS 2071 (GCA_900519085.1), *Y. bubula* strain CBS 12934 (GCA_900519075.1), *Y. alimentaria* strain CBS 10151 (GCA_900518985.1), *Yarrowia* sp. JCM 30695 (GCA_001602355.1), *Yarrowia* sp. JCM 30696 (GCA_001600535.1), *Yarrowia* sp. JCM 30694 (GCA_001600515.1) and *Y. keelungensis* JCM 14894 (GCA_001600195.1), as well as two representative strains of *Y. lipolytica* (*Y. lipolytica* CLIB122: GCF_000002525.2 and *Y. lipolytica* CLIB89: GCA_001761485.1). If not available, annotations for these genomes were prepared using AUGUSTUS with an included pre-computed training dataset of *Y. lipolytica*.

YLY mitochondrial genomes were downloaded from the NCBI and were compared to the mitochondria of other *Yarrowia* species [45] using NUCMER. We transferred annotations from *Y. alimentaria* mitochondrion (NC_016124.1) and *Y. phangngaensis* mitochondrion (NC_016126.1) to clade I mitochondria (C11 and E02) and annotations from *Y. lipolytica* mitochondrion [45] and *Y. galli* mitochondrion (NC_016116.1) to clade II mitochondria (B02, F05 and H10). Therefore, annotated genome features (rRNAs, tRNAs and all exons) from the published genomes were used as BLAST queries against the YLY mitochondria.

### Phenotypic characterization of strains

Phenotypic characterization was carried out using Biolog Phenotye MicroArrays (Biolog, Hayward, CA, USA) to determine the ability of YLY strains to utilize various substrates arrayed in microwell plates [63]. Phenotype MicroArrays (PM) measure the reduction of tetrazolium dye as a function of the redox energy produced during the oxidation of carbon and nitrogen sources or during exposure to specific osmolytes and pH conditions. Cellular metabolism resulting from the utilization of the substrates results in the formation of a purple color, which can be measured by spectrophotometry. Strains B02 and C11 were used as representative strains for assays and were cultured on PM1 and PM2A (carbon sources), PM3B (nitrogen sources), PM9 (osmolytes) and PM10 (pH) plates. Plates were inoculated according to the manufacturer’s instructions. All assays were performed in duplicate. Absorbance values at 590 and 750 nm were measured manually at regular intervals up to 55 h after the start of the experiment (except for strain B02 in plate PM1, where only one measurement was possible, hence a single time point after 48 h was used for one replicate). Absorbance was measured using an Infinite 200 spectrophotometer (Tecan, Männedorf, Switzerland). Differences between the endpoint reads (A590–A750), which corrects for background light scattering, were calculated and used for data analysis. Corrected absorbance values were averaged over all sample time points for each well and these values were converted to a score of 0–100. A probability density function for the scores was plotted using kernel density estimation with the *stats* package in R [64]. This was done separately for each plate and each strain to accommodate variation in absorbance values arising from differences in growth conditions across PM plates. The density function plots were manually inspected to locate probability density peaks and identify a threshold score value, with the assumption of a distribution of non-zero absorbance values for ‘negative’ wells in which the substrate was not utilized. If a well had a score higher than the threshold score, it was considered positive for substrate utilization [65] (**Additional File: Fig. S****5**).

### Analysis of genetic pathways

We used BLASTp v2.9.0 to screen the genetic repertoires of all five YLY strains against the databases ADP v3 [34], DRAMP v2.0 [35] and CAMP (version CAMPR 3) [36] for the detection of AMPs, and AntiSMASH v5.1.1 [37] to check for the production of antibiotics. The BLAST output was filtered to a minimal coverage of 0.5 and s minimal identity of 0.9.

## Supplementary Information

**Additional file 1: Table S1** Singleton genes of *Yarrowia*-like yeast genomes. Singleton genes were calculated with EDGAR [31]. Singletons are defined as genes without a reasonable BLAST hit against any gene within the other genomes in the comparison. We identified eight singletons for *Y. strain* C11, nine singletons for *Y. strain* E02 and one singleton for *Y. strain* H10.

**Additional file 2: Table S2** Retroviral-related Pol polyproteins identified in *Yarrowia*-like yeast genomes. We identified a total of 15 genes that are annotated as retrovirus-related Pol polyproteins within the genome of the five YLYs analyzed. The proteins belong to three different categories which are ‘Line-1’, ‘transposon 297’ and ‘opus’. While the clade II genomes of *Y.* strains B02, F05 and H10 do only encode for one such protein per genome, genomes of the clade I genomes of *Y.* strains C11 and E02 encode for several retrovirus-related Pol polyproteins. Thereby, it has to be noted that the type ‘opus’ is only present in the genome of strain E02 and that all respective genes belong to the set of singleton genes (*) of E02.

**Additional file 3: Table S3** ITS-D1/D2 regions of *Yarrowia*-like strains B02, C11, E02, F05 and H10. Numbers indicate contig (chromosome) (e.g. _2_) and position within the contig.

**Additional file 4: Figure S1** Coding density plots for *Yarrowia*-like yeast genomes. Predicted genes (including exons, introns and untranslated regions) were plotted to the chromosomal genomes of all five YLYs using DensityMap [62]. Regions with high density of genes are visualized in red, while the intergenic regions are shown in white. Clade II genomes have a higher density of coding sequences and less intergenic regions, while clade I genomes have longer intergenic regions.

**Additional file 5: Figure S2** Comparison of intergenic region lengths (top) and intron lengths (bottom) between clade I and clade II genomes. On the left, total and relative numbers of intergenic regions and introns are shown. Total and relative numbers of bases in these regions are shown on the right. Clade I genomes C11 and E02 (red) have relatively more large intergenic regions (> 1 kb) than clade II genomes B02, H10 and F05 (blue). This difference becomes even more significant in the number of total bases. Intergenic regions ≥1 kb in clade I genomes add up to 13 Mb, while the respective sum for clade II genomes only is 7.5 Mb. In clade I, 25% of intergenic bases are located within regions ≥10 kb, in clade II this number is only 3%. The difference in region numbers can also be shown for the introns, although a significant difference in intronic bases cannot be observed.

**Additional file 6: Figure S3** Number of reciprocal best BLAST hits between subsets of *Yarrowia*-like yeast genomes. Subsets were calculated with EDGAR [31] and visualized using R package UpSetR [28]. Species-specific genes should not be confused with ‘real’ singletons as listed in **Table S1**. Species-specific genes do not have reciprocal best BLAST hits in the other genomes; but ‘real’ singletons do not have reasonable BLAST hits against the other sets of genes, at all. **a** The common gene set of clade I genomes C11 and E02 includes 6237 (98.7%) genes. However, both genomes have ~ 80 genes without a bidirectional best BLAST hit. **b** The common gene set of clade II genomes B02, H10 and F02 includes 6424 (99.5%) genes. The number of species-specific genes is significantly smaller for clade I genomes. **c** The common gene set of the clade I strains C11 and E02 and strain B02 as a representative of clade II includes 5797 genes. This comparison shows that the overlap between C11 and E02 is much bigger than the overlap of these two strains with B02. Furthermore, B02 has more species-specific genes, which implies that gene sets of C11 and E02 are more similar than B02 compared to C11 or E02. **d** The common gene set of C11 (represents clade I) and B02 (represents clade II) along with *Y. lipolytica* CLIB122 consists of 5657 genes. Each of the strains has 362–421 species-specific genes. The numbers of genes shared between each set of genomes is also comparable. None of the strains seems to be closer related to one or the other strain. **e** The common gene set of B02, C11, E02 and *Y. lipolytica* CLIB122 includes 5630 genes. The number of species-specific genes for C11 and E02 is smallest. At the same time the overlay between these two strains is higher than the overlays with B02 or *Y. lipolytica* CLIB122. From these numbers we can conclude that strains C11 and E02 are more closely related to each other than any of the other strains within this comparison.

**Additional file 7: Figure S4** Positions of rDNA copies within the *Yarrowia-*like yeast nuclear genomes. Grey bars = scaffolds; dark blue arrows = full length rDNA copy; light blue arrows = partial rDNA sequence including ITS-D1/D2-region; white arrows = partial rDNA sequence without ITS-D1/D2-region; arrows pointing right = sequence on forward strand; arrows pointing left = sequence on reverse strand.

**Additional file 8: Figure S5** Phenotypic microarray assays. Growth curve parameters for each well for all phenotypic microarray (PM) plates were normalized to a PM score ranging from 0 to 100. A density histogram was plotted and a cutoff value (red line) was identified by fitting a density function for the bimodal distribution to identify two peaks for substrates that were not utilized and for substrates that were utilized. If the score for each well was higher than the cutoff (PM score > 7), a substrate was concluded to be utilized by the yeast strain.

## Data Availability

The genome sequences of YLY strains B02, C11 and E02 were deposited as whole-genome shotgun projects at DDBJ/ENA/GenBank with accessions JAAQQV000000000 (https://www.ncbi.nlm.nih.gov/nuccore/JAAQQV000000000), JAAQQW000000000 (https://www.ncbi.nlm.nih.gov/nuccore/JAAQQW000000000) and JAAQQX000000000 (https://www.ncbi.nlm.nih.gov/nuccore/JAAQQX000000000), respectively. The versions described in this paper are versions JAAQQV010000000, JAAQQW010000000 and JAAQQX010000000. The short-read data have been deposited in the European Nucleotide Archive (ENA) with the following sample accession numbers: ERS5524537-ERS5524546. The complete study can also be accessed directly using the following URL: https://www.ebi.ac.uk/ena/data/view/PRJEB42422. Whole-genome sequences and mitochondrial sequences of reference strains were downloaded from NCBI as GenBank files and are available with accessions GCA_900518995.1, GCA_900519025.1, GCA_900519005.1, GCA_900519015.1, GCA_900519065.1, GCA_900519055.1, GCA_900519045.1, GCA_900519085.1, GCA_900519075.1, GCA_900518985.1, GCA_001602355.1, GCA_001600535.1, GCA_001600515.1, GCA_001600195.1, GCF_000002525.2, GCA_001761485.1, NC_016124.1, NC_016126.1 and NC_016116.1.

## Abbreviations

rRNA/rDNA: Ribosomal RNA/ribosomal DNA
YLY: *Yarrowia*-like yeast
AMP: Antimicrobial peptide
LSU: Large subunit
RNA-Seq: RNA sequencing
ITS: Internal transcribed spacer
SNP: Single nucleotide polymorphism
indels: Insertions and deletions
PM: Phenotype microarray
NRPS: Non-ribosomal peptide synthetase
YEME: Yeast extract/malt extract
TE tris: EDTA buffer
