# Supplementary material for: Genomic analysis of novel Yarrowia-like yeast symbionts associated with the carrion-feeding burying beetle Nicrophorus vespilloides
Source: BMC Genomics. 2021 May 3;22:323. doi: 10.1186/s12864-021-07597-z (PMC8091737; doi:10.1186/s12864-021-07597-z)
Supplement: Supplementary file 1 — Additional file 1: Table S1 Singleton genes of Yarrowia-like yeast genomes. Singleton genes were calculated with EDGAR [31]. Singletons are defined as genes without a reasonable BLAST hit against any gene within the other genomes in the comparison. We identified eight singletons for Y. strain C11, nine singletons for Y. strain E02 and one singleton for Y. strain H10. [file 12864_2021_7597_MOESM1_ESM.docx]

**Table S1** Singleton genes of *Yarrowia*-like genomes. Singleton genes were calculated with EDGAR [41].

| **Singleton** | **Annotation** | **Similar sequence present in other genomes?** |
| --- | --- | --- |
| C11.g1810 | Hpyothetical protein | YES and NO: first third of the gene not present in E02, rest of the gene 100% ID in E02 |
| C11.g2523 | Hpyothetical protein | NO |
| C11.g3995 | Hpyothetical protein | YES and NO: 1. half of the gene not present in E02, 2. half of the gene 100% ID in E02 |
| C11.g6097 | Hpyothetical protein | YES: 99% ID with E02; 5 SNPs in E02 🡪 no gene predicted for E02 at respective position |
| C11.g6142 | Hpyothetical protein | YES: 2x in E02, but different genes annotated at respective positions |
| C11.g6218 | Hpyothetical protein | YES: 99% ID with E02; 4 SNPs in E02 🡪 no gene predicted for E02 at respective position |
| C11.g6235 | Hpyothetical protein | YES: 97% ID with E02; 9 SNPs in E02 🡪 no gene predicted for E02 at respective position |
| C11.g6238 | Hpyothetical protein | YES: 99% ID with E02; 2 SNPs in E02 🡪 no gene predicted for E02 at respective position |
| E02.g523 | Hpyothetical protein | YES: 99% ID with C11, 2 SNPs and 2 deletion in C11 🡪 no gene predicted for C11 at respective position |
| E02.g2210 | Hpyothetical protein | NO |
| E02.g2211 | Retrovirus-related Pol polyprotein from opus | NO |
| E02.g4884 | Retrovirus-related Pol polyprotein from opus | NO |
| E02.g5804 | Retrovirus-related Pol polyprotein from opus | NO |
| E02.g6018 | Retrovirus-related Pol polyprotein from opus | NO |
| E02.g6255 | Hpyothetical protein | YES: 99% ID with C11; 2 SNPs in C11 🡪 no gene predicted for C11 at respective position |
| E02.g6256 | Hpyothetical protein | Yes: 96% ID with C11, several SNPs and InDels 🡪 no gene predicted for C11 at respective position |
| E02.g6291 | Hpyothetical protein | NO |
| H10.g4568 | Hpyothetical protein | YES: H10.g4568 + H10.g4567 ≙ F05.3968 ≙ B02.g1672 |

Singletons are defined as genes without a reasonable BLAST hit against any gene within the other genomes in the comparison. We identified eight singletons for *Y. strain* C11, nine singletons for *Y. strain* E02 and one singleton for *Y. strain* H10.
