# Supplementary material for: Genomic analysis of novel Yarrowia-like yeast symbionts associated with the carrion-feeding burying beetle Nicrophorus vespilloides
Source: BMC Genomics. 2021 May 3;22:323. doi: 10.1186/s12864-021-07597-z (PMC8091737; doi:10.1186/s12864-021-07597-z)
Supplement: Supplementary file 2 — Additional file 2: Table S2 Retroviral-related Pol polyproteins identified in Yarrowia-like yeast genomes. We identified a total of 15 genes that are annotated as retrovirus-related Pol polyproteins within the genome of the five YLYs analyzed. The proteins belong to three different categories which are ‘Line-1’, ‘transposon 297’ and ‘opus’. While the clade II genomes of Y. strains B02, F05 and H10 do only encode for one such protein per genome, genomes of the clade I genomes of Y. strains C11 and E02 encode for several retrovirus-related Pol polyproteins. Thereby, it has to be noted that the type ‘opus’ is only present in the genome of strain E02 and that all respective genes belong to the set of singleton genes (*) of E02. [file 12864_2021_7597_MOESM2_ESM.docx]

**Table S2** Retroviral-related Pol polyproteins identified for *Yarrowia*-like genomes.

|  |  |  | **Clade I** | | **Clade II** | | |
| --- | --- | --- | --- | --- | --- | --- | --- |
| **Description** | **EC number** | **Gene product** | **C11** | **E02** | **B02** | **F05** | **H10** |
|  |  |  | **Gene ID** | **Gene ID** | **Gene ID** | **Gene ID** | **Gene ID** |
| Retrovirus-related Pol polyprotein from opus | 3.4.23.- / 2.7.7.49 / - | Protease / Reverse transcriptase / Endonuclease | - | E02.g2211* | - | - | - |
| Retrovirus-related Pol polyprotein from opus | 3.4.23.- / 2.7.7.49 / - | Protease / Reverse transcriptase / Endonuclease | - | E02.g4884* | - | - | - |
| Retrovirus-related Pol polyprotein from opus | 3.4.23.- / 2.7.7.49 / - | Protease / Reverse transcriptase / Endonuclease | - | E02.g5804* | - | - | - |
| Retrovirus-related Pol polyprotein from opus | 3.4.23.- / 2.7.7.49 / - | Protease / Reverse transcriptase / Endonuclease | - | E02.g6018* | - | - | - |
| Retrovirus-related Pol polyprotein from transposon 297 | 3.4.23.- / 2.7.7.49 / - | Protease / Reverse transcriptase / Endonuclease | C11.g3496 | E02.g3690 | B02.g3007 | F05.g2843 | H10.g5902 |
| Retrovirus-related Pol polyprotein Line-1 | 2.7.7.49 / 3.1.21.- | Reverse transcriptase / Endonuclease | C11.g1197 | E02.g4665 | - | - | - |
| Retrovirus-related Pol polyprotein Line-1 | 2.7.7.49 / 3.1.21.- | Reverse transcriptase / Endonuclease | C11.g1198 | E02.g4666 | - | - | - |
| Retrovirus-related Pol polyprotein Line-1 | 2.7.7.49 / 3.1.21.- | Reverse transcriptase / Endonuclease | C11.g1665 | E02.g5133 | - | - | - |
| Retrovirus-related Pol polyprotein Line-1 | 2.7.7.49 / 3.1.21.- | Reverse transcriptase / Endonuclease | C11.g1971 | - | - | - | - |
| Retrovirus-related Pol polyprotein Line-1 | 2.7.7.49 / 3.1.21.- | Reverse transcriptase / Endonuclease | C11.g4394 | E02.g2537 | - | - | - |
| Retrovirus-related Pol polyprotein Line-1 | 2.7.7.49 / 3.1.21.- | Reverse transcriptase / Endonuclease | C11.g6045 | - | - | - | - |
| Retrovirus-related Pol polyprotein Line-1 | 2.7.7.49 / 3.1.21.- | Reverse transcriptase / Endonuclease | - | E02.g52 | - | - | - |
| Retrovirus-related Pol polyprotein Line-1 | 2.7.7.49 / 3.1.21.- | Reverse transcriptase / Endonuclease | - | E02.g887 | - | - | - |
| Retrovirus-related Pol polyprotein TNT 1-94 | 3.4.23.- / 2.7.7.49 / - | Protease / Reverse transcriptase / Endonuclease | C11.g2733 | E02.g2326 | - | - | - |
| Retrovirus-related Pol polyprotein TNT 1-94 | 3.4.23.- / 2.7.7.49 / - | Protease / Reverse transcriptase / Endonuclease | C11.g5503 | - | - | - | - |

We identified a total of 15 genes that are annotated as retrovirus-related Pol polyproteins within the genome of the five YLYs analyzed. The proteins belong to three different categories which are ‘Line-1’, ‘transposon 297’ and ‘opus’. While the Clade II genomes of *Y.* strains B02, F05 and H10 do only encode for one such protein per genome, genomes of the Clade I genomes of *Y.* strains C11 and E02 encode for several retrovirus-related Pol polyproteins. Thereby, it has to be noted that the type ‘opus’ is only present in the genome of strain E02 and that all respective genes belong to the set of singleton genes (*) of E02.
