# Supplementary figures and images for: Genomic analysis of novel Yarrowia-like yeast symbionts associated with the carrion-feeding burying beetle Nicrophorus vespilloides
Source: BMC Genomics. 2021 May 3;22:323. doi: 10.1186/s12864-021-07597-z (PMC8091737; doi:10.1186/s12864-021-07597-z)

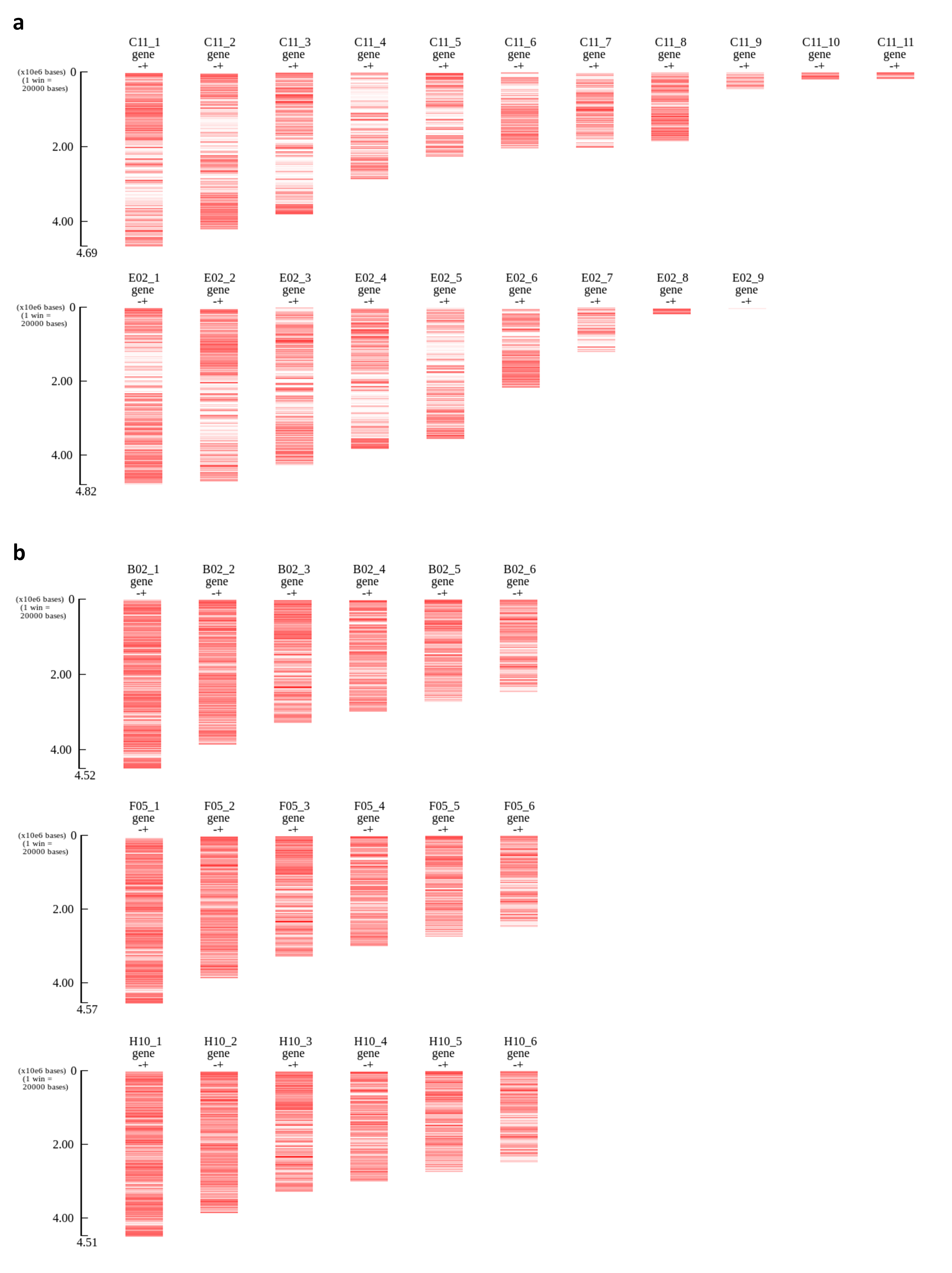

Supplement: Supplementary file 4 — Additional file 4: Figure S1 Coding density plots for Yarrowia-like yeast genomes. Predicted genes (including exons, introns and untranslated regions) were plotted to the chromosomal genomes of all five YLYs using DensityMap [62]. Regions with high density of genes are visualized in red, while the intergenic regions are shown in white. Clade II genomes have a higher density of coding sequences and less intergenic regions, while clade I genomes have longer intergenic regions. [file 12864_2021_7597_MOESM4_ESM.png]

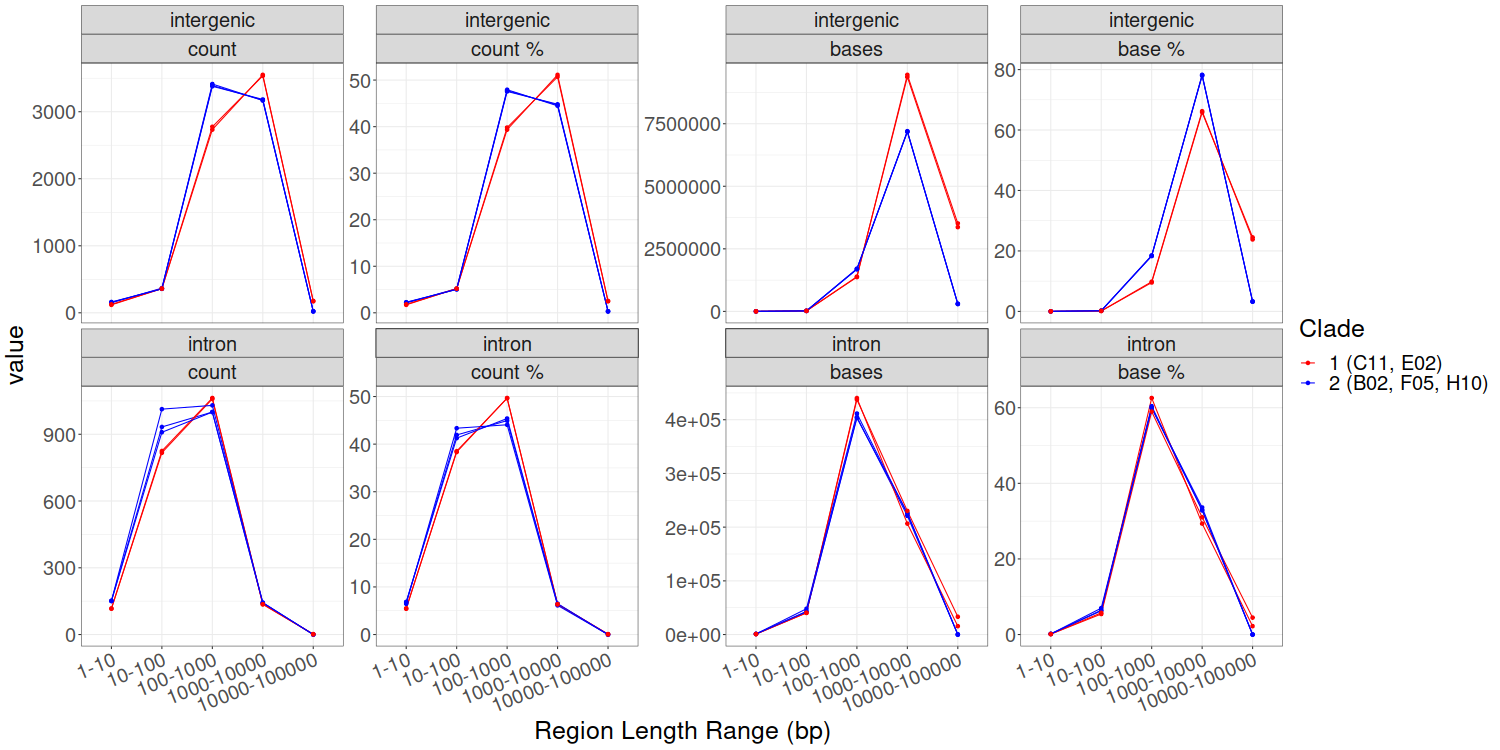

Supplement: Supplementary file 5 — Additional file 5: Figure S2 Comparison of intergenic region lengths (top) and intron lengths (bottom) between clade I and clade II genomes. On the left, total and relative numbers of intergenic regions and introns are shown. Total and relative numbers of bases in these regions are shown on the right. Clade I genomes C11 and E02 (red) have relatively more large intergenic regions (> 1 kb) than clade II genomes B02, H10 and F05 (blue). This difference becomes even more significant in the number of total bases. Intergenic regions ≥1 kb in clade I genomes add up to 13 Mb, while the respective sum for clade II genomes only is 7.5 Mb. In clade I, 25% of intergenic bases are located within regions ≥10 kb, in clade II this number is only 3%. The difference in region numbers can also be shown for the introns, although a significant difference in intronic bases cannot be observed. [file 12864_2021_7597_MOESM5_ESM.png]

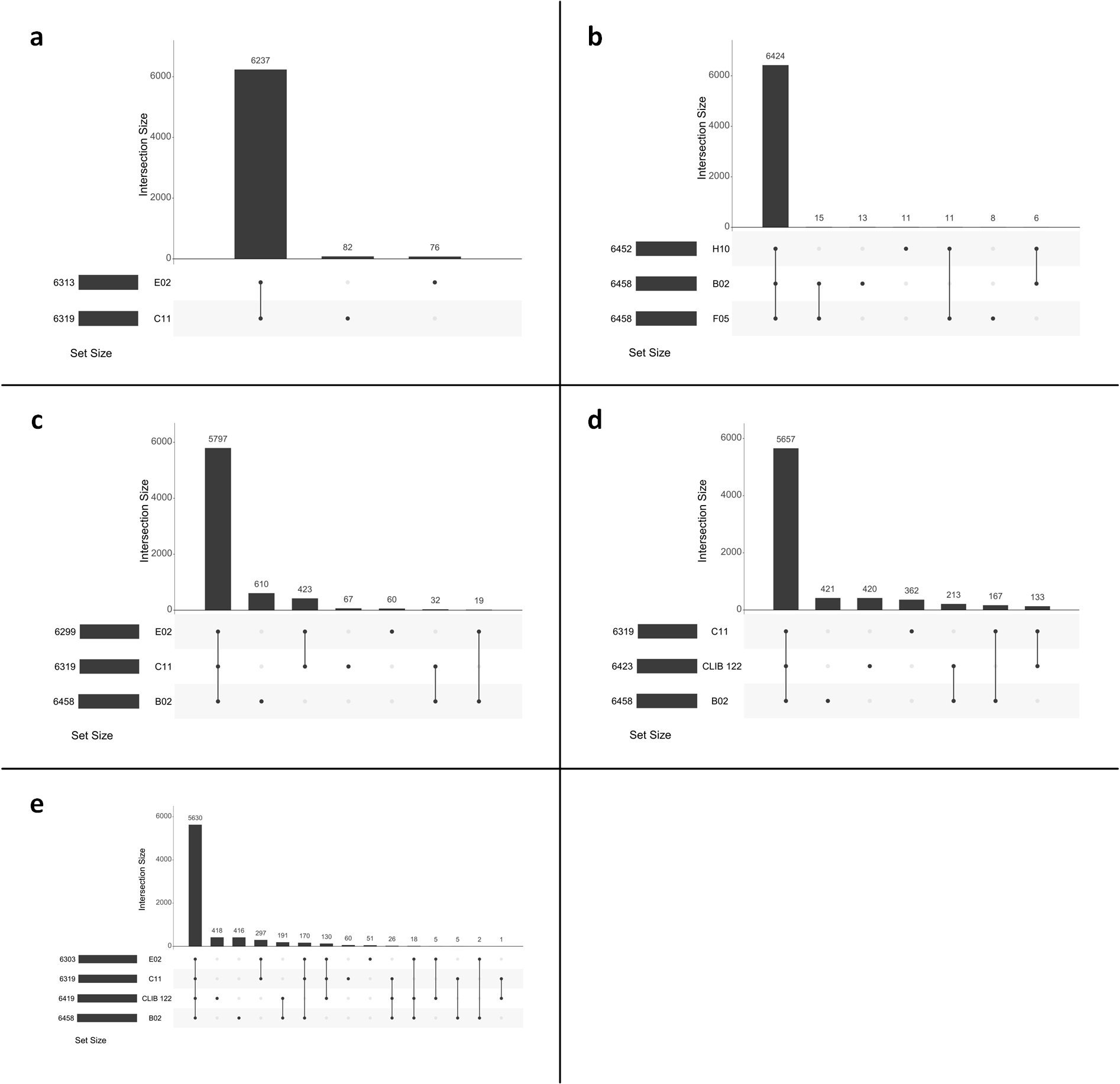

Supplement: Supplementary file 6 — Additional file 6: Figure S3 Number of reciprocal best BLAST hits between subsets of Yarrowia-like yeast genomes. Subsets were calculated with EDGAR [31] and visualized using R package UpSetR [28]. Species-specific genes should not be confused with ‘real’ singletons as listed in Table S1. Species-specific genes do not have reciprocal best BLAST hits in the other genomes; but ‘real’ singletons do not have reasonable BLAST hits against the other sets of genes, at all. a The common gene set of clade I genomes C11 and E02 includes 6237 (98.7%) genes. However, both genomes have ~ 80 genes without a bidirectional best BLAST hit. b The common gene set of clade II genomes B02, H10 and F02 includes 6424 (99.5%) genes. The number of species-specific genes is significantly smaller for clade I genomes. c The common gene set of the clade I strains C11 and E02 and strain B02 as a representative of clade II includes 5797 genes. This comparison shows that the overlap between C11 and E02 is much bigger than the overlap of these two strains with B02. Furthermore, B02 has more species-specific genes, which implies that gene sets of C11 and E02 are more similar than B02 compared to C11 or E02. d The common gene set of C11 (represents clade I) and B02 (represents clade II) along with Y. lipolytica CLIB122 consists of 5657 genes. Each of the strains has 362–421 species-specific genes. The numbers of genes shared between each set of genomes is also comparable. None of the strains seems to be closer related to one or the other strain. e The common gene set of B02, C11, E02 and Y. lipolytica CLIB122 includes 5630 genes. The number of species-specific genes for C11 and E02 is smallest. At the same time the overlay between these two strains is higher than the overlays with B02 or Y. lipolytica CLIB122. From these numbers we can conclude that strains C11 and E02 are more closely related to each other than any of the other strains within this comparison. [file 12864_2021_7597_MOESM6_ESM.zip › Figure_S3_V2.png]

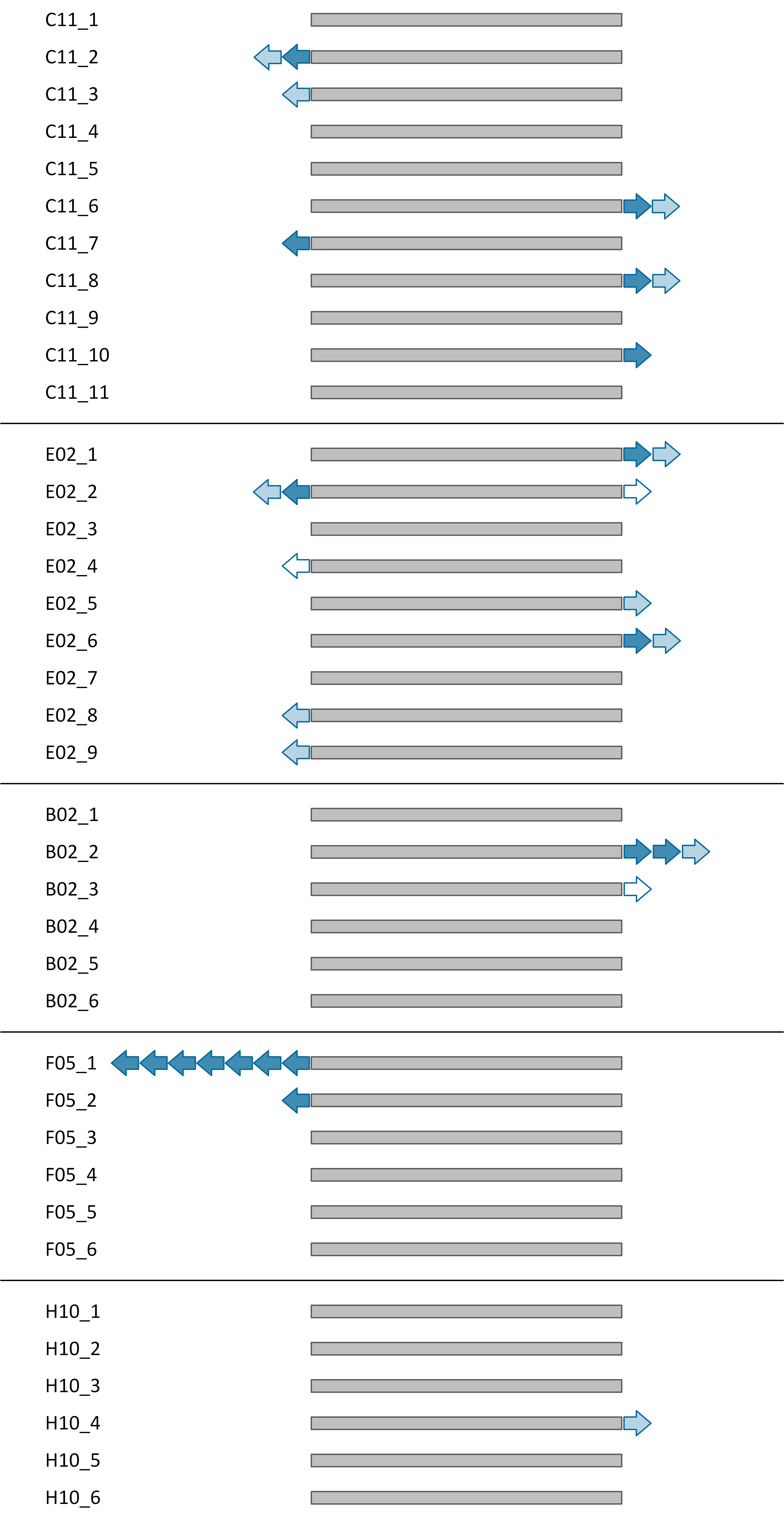

Supplement: Supplementary file 7 — Additional file 7: Figure S4 Positions of rDNA copies within the Yarrowia-like yeast nuclear genomes. Grey bars = scaffolds; dark blue arrows = full length rDNA copy; light blue arrows = partial rDNA sequence including ITS-D1/D2-region; white arrows = partial rDNA sequence without ITS-D1/D2-region; arrows pointing right = sequence on forward strand; arrows pointing left = sequence on reverse strand. [file 12864_2021_7597_MOESM7_ESM.png]

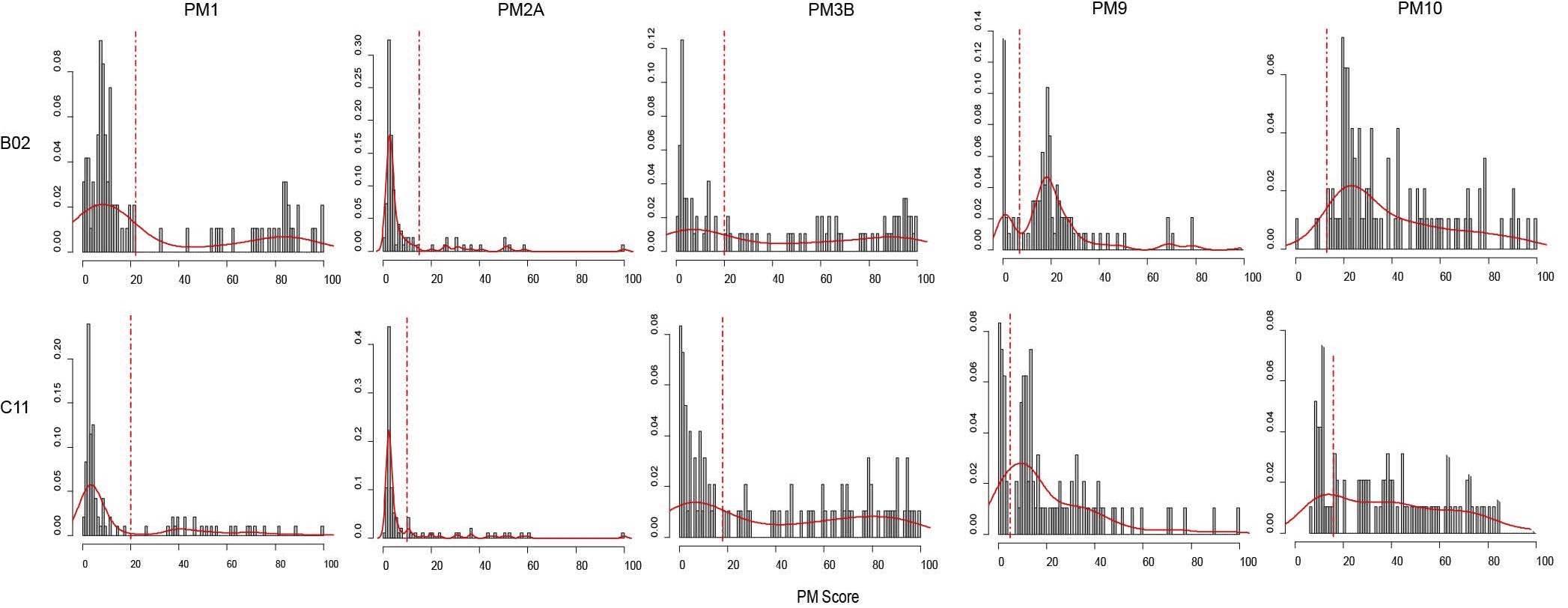

Supplement: Supplementary file 8 — Additional file 8: Figure S5 Phenotypic microarray assays. Growth curve parameters for each well for all phenotypic microarray (PM) plates were normalized to a PM score ranging from 0 to 100. A density histogram was plotted and a cutoff value (red line) was identified by fitting a density function for the bimodal distribution to identify two peaks for substrates that were not utilized and for substrates that were utilized. If the score for each well was higher than the cutoff (PM score > 7), a substrate was concluded to be utilized by the yeast strain. [file 12864_2021_7597_MOESM8_ESM.jpg]
