# Supplementary material for: Genomic analysis of novel Yarrowia-like yeast symbionts associated with the carrion-feeding burying beetle Nicrophorus vespilloides
Source: BMC Genomics. 2021 May 3;22:323. doi: 10.1186/s12864-021-07597-z (PMC8091737; doi:10.1186/s12864-021-07597-z)
Supplement: Supplementary file 6 — Additional file 6: Figure S3 Number of reciprocal best BLAST hits between subsets of Yarrowia-like yeast genomes. Subsets were calculated with EDGAR [31] and visualized using R package UpSetR [28]. Species-specific genes should not be confused with ‘real’ singletons as listed in Table S1. Species-specific genes do not have reciprocal best BLAST hits in the other genomes; but ‘real’ singletons do not have reasonable BLAST hits against the other sets of genes, at all. a The common gene set of clade I genomes C11 and E02 includes 6237 (98.7%) genes. However, both genomes have ~ 80 genes without a bidirectional best BLAST hit. b The common gene set of clade II genomes B02, H10 and F02 includes 6424 (99.5%) genes. The number of species-specific genes is significantly smaller for clade I genomes. c The common gene set of the clade I strains C11 and E02 and strain B02 as a representative of clade II includes 5797 genes. This comparison shows that the overlap between C11 and E02 is much bigger than the overlap of these two strains with B02. Furthermore, B02 has more species-specific genes, which implies that gene sets of C11 and E02 are more similar than B02 compared to C11 or E02. d The common gene set of C11 (represents clade I) and B02 (represents clade II) along with Y. lipolytica CLIB122 consists of 5657 genes. Each of the strains has 362–421 species-specific genes. The numbers of genes shared between each set of genomes is also comparable. None of the strains seems to be closer related to one or the other strain. e The common gene set of B02, C11, E02 and Y. lipolytica CLIB122 includes 5630 genes. The number of species-specific genes for C11 and E02 is smallest. At the same time the overlay between these two strains is higher than the overlays with B02 or Y. lipolytica CLIB122. From these numbers we can conclude that strains C11 and E02 are more closely related to each other than any of the other strains within this comparison. [file 12864_2021_7597_MOESM6_ESM.zip › Addcorr1.pdf]

Dear Warjun S. Dagunton,

must have been a problem between windows and linux. The respective file is a .txt - which should be fine considering the intructions for authors.

I made moderate changes to the file which only affects line breaks... not altering the content of the file. I have attatched it to this email. I hope you can access the file now? Please let me know if you want me to upload the file somewhere or if this email-attatchment is fine.

Best regards, Karina Brinkrolf
